# Loss of Function of Intestinal IL-17 and IL-22 Producing Cells Contributes to Inflammation and Viral Persistence in SIV-Infected Rhesus Macaques

**DOI:** 10.1371/journal.ppat.1005412

**Published:** 2016-02-01

**Authors:** Emily S. Ryan, Luca Micci, Rémi Fromentin, Sara Paganini, Colleen S. McGary, Kirk Easley, Nicolas Chomont, Mirko Paiardini

**Affiliations:** 1 Division of Microbiology & Immunology, Yerkes National Primate Research Center, Emory University School of Medicine, Atlanta, Georgia, United States of America; 2 Department of Microbiology, Infectiology and Immunology, Université de Montréal, Faculty of Medicine, and Centre de Recherche du CHUM, Montreal, Québec, Canada; 3 Department of Biostatistics & Bioinformatics, Rollins School of Public Health, Emory University, Atlanta, Georgia, United States of America; 4 Department of Pathology and Laboratory Medicine, Emory University School of Medicine, Atlanta, Georgia, United States of America; National Institutes of Health, UNITED STATES

## Abstract

In HIV/SIV-infected humans and rhesus macaques (RMs), a severe depletion of intestinal CD4^+^ T-cells producing interleukin IL-17 and IL-22 associates with loss of mucosal integrity and chronic immune activation. However, little is known about the function of IL-17 and IL-22 producing cells during lentiviral infections. Here, we longitudinally determined the levels and functions of IL-17, IL-22 and IL-17/IL-22 producing CD4^+^ T-cells in blood, lymph node and colorectum of SIV-infected RMs, as well as how they recover during effective ART and are affected by ART interruption. Intestinal IL-17 and IL-22 producing CD4^+^ T-cells are polyfunctional in SIV-uninfected RMs, with the large majority of cells producing four or five cytokines. SIV infection induced a severe dysfunction of colorectal IL-17, IL-22 and IL-17/IL-22 producing CD4^+^ T-cells, the extent of which associated with the levels of immune activation (HLA-DR^+^CD38^+^), proliferation (Ki-67^+^) and CD4^+^ T-cell counts before and during ART. Additionally, Th17 cell function during ART negatively correlated with residual plasma viremia and levels of sCD163, a soluble marker of inflammation and disease progression. Furthermore, IL-17 and IL-22 producing cell frequency and function at various pre, on, and off-ART experimental points associated with and predicted total SIV-DNA content in the colorectum and blood. While ART restored Th22 cell function to levels similar to pre-infection, it did not fully restore Th17 cell function, and all cell types were rapidly and severely affected—both quantitatively and qualitatively—after ART interruption. In conclusion, intestinal IL-17 producing cell function is severely impaired by SIV infection, not fully normalized despite effective ART, and strongly associates with inflammation as well as SIV persistence off and on ART. As such, strategies able to preserve and/or regenerate the functions of these CD4^+^ T-cells central for mucosal immunity are critically needed in future HIV cure research.

## Author Summary

Persistent immune activation and inflammation are key features and strong predictors of morbidity/mortality in HIV infection. A specific quantitative loss of Th17 and Th22 CD4^+^ T-cells, which are crucial to maintaining the mucosal immunity, has been shown to directly associate with microbial translocation, systemic immune activation, and disease progression. Despite this, how HIV infection impacts Th17 and Th22 cell qualitative function remains largely unknown. To address this important question, we investigated Th17 and Th22 cell function and levels longitudinally before, during, and after ART in the rhesus macaque model of SIV infection in the colorectum, blood, and lymph node. We found that mucosal Th17 and Th22 cell function and levels were profoundly ablated upon SIV infection, and only partially restored by ART. Importantly, this loss of IL-17 and IL-22 producing cell function directly correlated with disease progression, immune activation, and SIV persistence. These data strongly support a molecular link between persistent inflammation and viral persistence as well as the importance of preserving intestinal Th17 and Th22 cell function during HIV infection, and urge the need for therapeutic strategies aimed at improving these cells function in future HIV cure research.

## Introduction

HIV infection in humans and SIV infection in rhesus macaques (RMs) is characterized by the establishment of high and persistent levels of immune activation and inflammation, which are strong and independent predictors of disease progression in the natural history of infection and co-morbidities/mortalities in individuals on antiretroviral therapy (ART). While the causes of this sustained immune activation during chronic HIV/SIV infections are complex and not completely understood, the severe depletion of intestinal CD4^+^ T-cells early after infection and the associated loss of mucosal barrier integrity are commonly regarded as two of the most critical contributors to persistent immune activation and disease progression [1–4].

CD4^+^ T-cells, the main targets of HIV and SIV infections, can be classified in subsets of Th1, Th2, Th17, Th22, follicular helper (Tfh), and regulatory T-cells (Treg) based on their phenotypes, cytokine production, transcriptional profiles and anatomic localization [5,6]. Th17 cells are characterized by the expression of CCR6 and the transcription factor RORγt, as well as by the production of IL-17 [7–13]. Th22 cells are characterized by the expression of the chemokine receptors CCR4, CCR6, and CCR10, as well as the transcription factor aryl hydrocarbon receptor (AHR) [14–17]. The main cell targets of IL-22 are mucosal epithelial cells [18–20]. The *in vivo* effector functions of IL-17 and IL-22 are crucial to maintaining mucosal immunity against specific pathogens and include the recruitment of neutrophils to the sites of bacterial invasion, the enhancement of mucosal barrier repair and maintenance through stimulation of epithelial cell proliferation and tight junction protein production, as well as the induction of antimicrobial proteins, including beta-defensin [18,19,21,22]. Indeed, IL-17 and/or IL-22 associated protection has been described for numerous infections, including *Citrobacter rodentium* [23], *Klebsiella pneumonia* [24], *Toxoplasma gondii* [25], *Candida albicans* [26], Bordetella pertussis [27], Pneumocystis carinii [28], among others.

Intestinal IL-17 and IL-22 producing cells are preferentially depleted in chronically HIV/SIV-infected subjects, with the severity of their depletion correlating with the extent of microbial translocation, chronic immune activation, and disease progression [2,29–36], as well as with effective CD4^+^ T-cell restoration in gut-associated lymphoid tissue of HIV-infected patients on ART [37]. Further supporting their roles in disease progression, IL-17 and IL-22 producing CD4^+^ T-cells have been shown to be relatively preserved in the gastrointestinal tracts of chronically SIV-infected sooty mangabeys (SMs) and African green monkeys [29,34,38,39], natural hosts of SIV infection that avoid microbial translocation, chronic immune activation and progression to AIDS, as well as in HIV controllers and long-term nonprogressors [40–42]. In addition, a recent study showed that the size of the Th17 cell compartment prior to infection limited viral replication in SIV-infected RMs [43].

Although several studies have confirmed the loss of intestinal IL-17 producing CD4^+^ T-cells, the dynamics of IL-22 and IL-17/IL-22 producing T-cells during HIV and SIV infection have not been studied extensively. In addition, very little is known about how pathogenic HIV/SIV infection also impacts the functional ability of Th17 and Th22 cells to co-produce additional cytokines. This is an important issue, since the ability of individual T-cells to simultaneously perform multiple effector functions is critical for protective immune responses against pathogens [22]. Recently it was shown in HIV infected individuals that intestinal Th17 cell function, which was assessed for coproduction of IFNγ, TNFα, and IL-22, independently predicted immune activation [44]. In addition, there were two very recent studies connecting the initiation of early ART therapy to preserved Th17 function and reversed HIV-induced immune activation [45,46]. Since the existing functional studies have been in solely HIV-infected humans, we investigated intestinal Th17 cell function in the context of SIV infection in RMs to potentially support and further these results. In this study, we used SIV-infected RMs to investigate the levels and functions of blood and tissue IL-17, IL-22, and IL-17/IL-22 producing CD4^+^ T-cells during progressive SIV infection, how these features are recovered during effective antiretroviral therapy and affected by structured ART interruption, as well as how they associate with immune activation and viral persistence.

## Results

### Study design and assessment of IL-17 and IL-22 producing CD4^+^ T-cell polyfunctionality in RMs

We first aimed to determine the ability of blood and tissue-derived IL-17 and IL-22 producing CD4^+^ T-cells to co-produce multiple cytokines in RMs by using a five-cytokine flow cytometric panel that included IL-17, IL-22, IFN-γ, TNF-α, and IL-2. Co-production of these cytokines after PMA and Ionomycin stimulation was assessed through Boolean gating in live (dead cells were excluded based on live/dead staining) CD3^+^CD4^+^ T-cells producing IL-17, IL-22 or both cytokines (IL-17/IL-22; Th17/Th22) in blood, lymph node (LN) and colorectum. Since those cytokines are not produced by naïve CD4^+^ T-cells, our gating strategies correct for differences in proportions of naïve/memory CD4^+^ T-cells between PBMC, LN and MMC.

**Fig 1:**
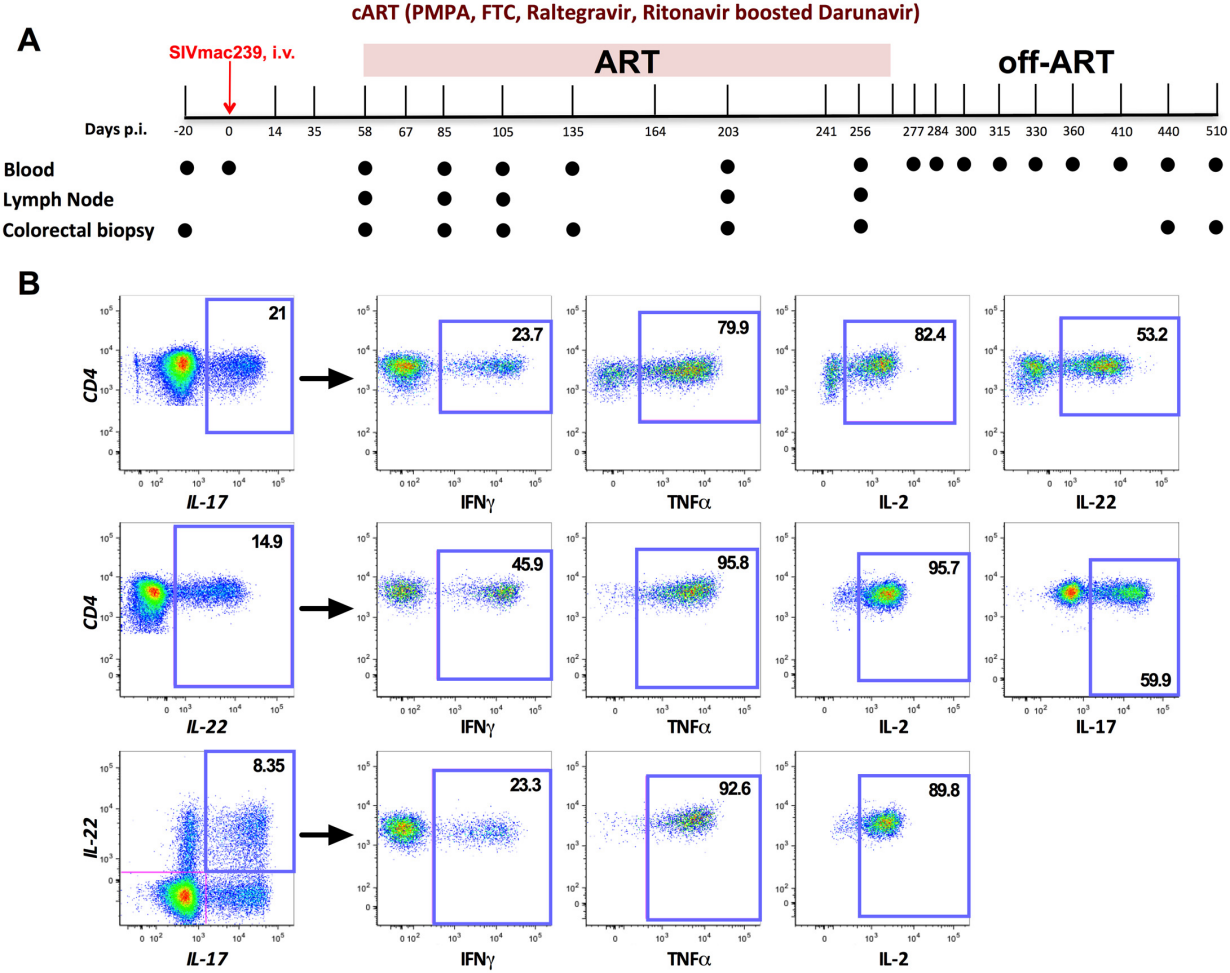
Study timeline and representative cytokine panel staining. **(A)** Complete study timeline of longitudinal tissue collections, SIVmac239 i.v. infection (d.0 p.i.), ART treatment (d. 58 to d.256 p.i.), and off-ART time period. **(B)** Representative staining for IL-17, IL-22, IFNγ, TNFα, and IL-2 within intestinal CD4^+^ T-cells in a representative uninfected RM.

A representative staining for the different cytokines is shown in Fig 1B. In determining functionality of these cells, we used two different methods of analysis. First, we used the SPICE program to plot the comprehensive cytokine expression profiles of each cell subset for each time point investigated. Secondly, we assigned each cell subset at each time point a “functional score”, being the mean number of cytokines produced per individual cell. Through this functional score analysis, we could then effectively quantify cell subset function, as well as correlate this with the numerous virologic and immunologic parameters of SIV infection investigated in the study. Throughout this manuscript, the words “cytokine profile” refer to a result of the SPICE analysis, and “functional score” refers to the mean number of cytokines produced by an individual cell. The functional score analysis paired with the SPICE cytokine profile analysis allowed us to assess IL-17 and IL-22 producing CD4^+^ T-cell polyfunctionality longitudinally before SIV infection (with the exception of the lymph node), during its untreated early phase (day 58 p.i.), and at time points throughout ART treatment, as well as after interruption (Fig 1A).

### The function of IL-17 and IL-22 producing CD4^+^ T-cells is higher in colorectum than blood of SIV-uninfected RMs

**Fig 2:**
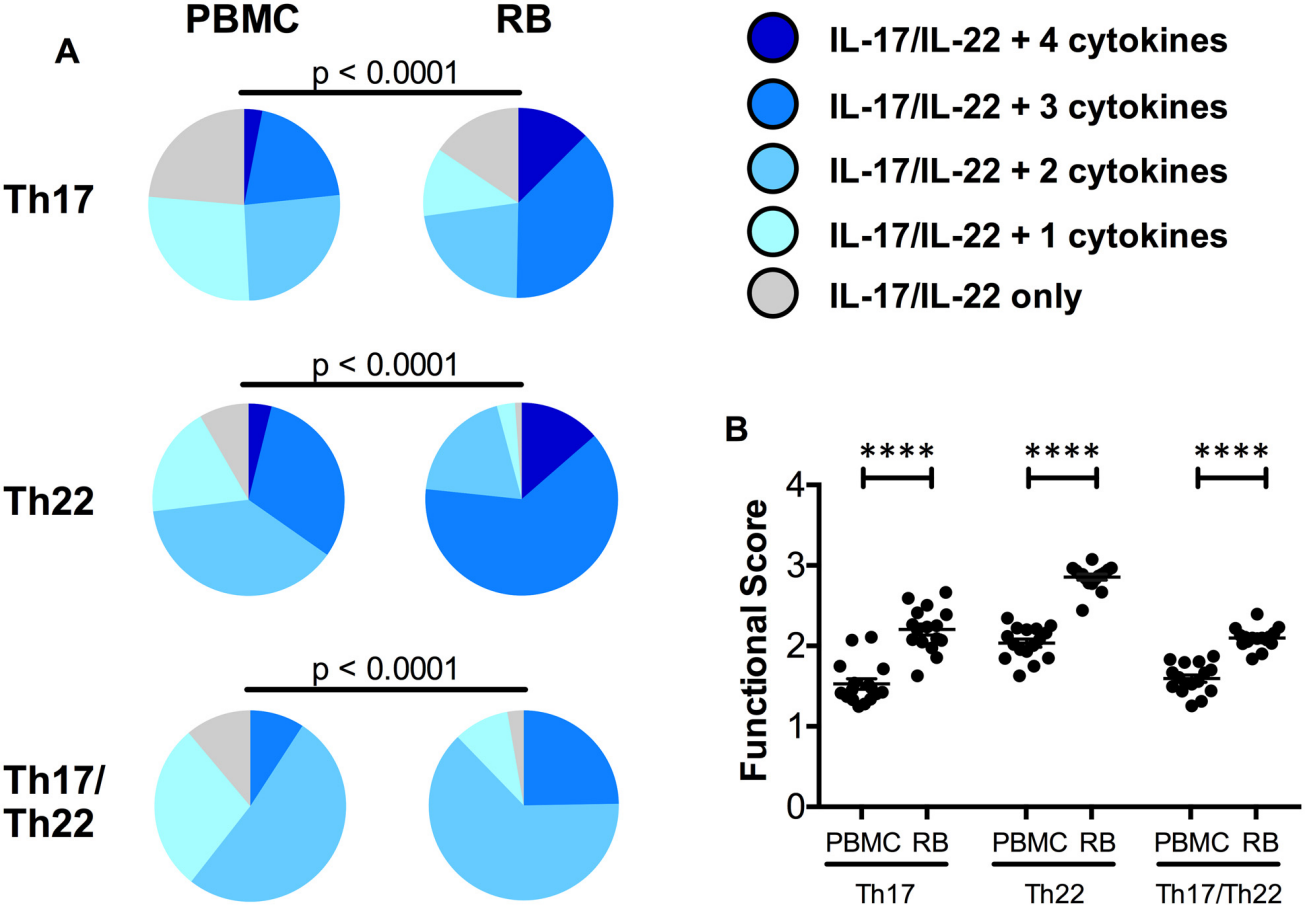
IL-17 producing cell function is higher in colorectum than blood in uninfected RMs. **(A)** Comparison between cytokine profiles of Th17, Th22, and Th17/Th22 cells in PBMC and RB in uninfected RMs (d. -20 p.i.). Cytokine profiles were generated for each cell population by SPICE program v. 5.33, and were calculated by Flowjo Boolean gating. **(B)** Functional scores were compared for all three subsets between PBMC and RB. Functional scores represent average number of cytokines produced per individual cell (see Methods). Averaged data are presented as means ± SEM.

We first investigated if the function of IL-17 and IL-22 producing cells is different at the mucosal level as compared to blood in the 16 SIV-uninfected control RMs included in this first section of the study. As shown in Fig 2, colorectal IL-17, IL-22, and IL-17/IL-22 producing CD4^+^ T-cell cytokine profiles were significantly different from their blood counterparts. Specifically, all three intestinal CD4^+^ T-cell subsets showed remarkably higher fractions of cells producing four and five cytokines (Fig 2A; p<0.0001 for all) and significantly higher functional scores (2.2 ± 0.07; 2.9 ± 0.04; 2.1 ± 0.03 for Th17, Th22, and Th17/Th22, respectively) than the cells in the blood (1.5 ± 0.06; 1.9 ± 0.04; 1.6 ± 0.05 for Th17, Th22, and Th17/Th22, respectively) of the same animals (Fig 2B; p<0.0001 for all).

Thus, consistent with their important roles in antimicrobial immunity and mucosal integrity [22], IL-17 and IL-22 producing CD4^+^ T-cells acquire much higher polyfunctional profiles when present in the gastrointestinal tract.

### SIV-infection severely reduces the frequencies and alters the function of intestinal IL-17 and IL-22 producing CD4^+^ T-cells in RMs

**Fig 3:**
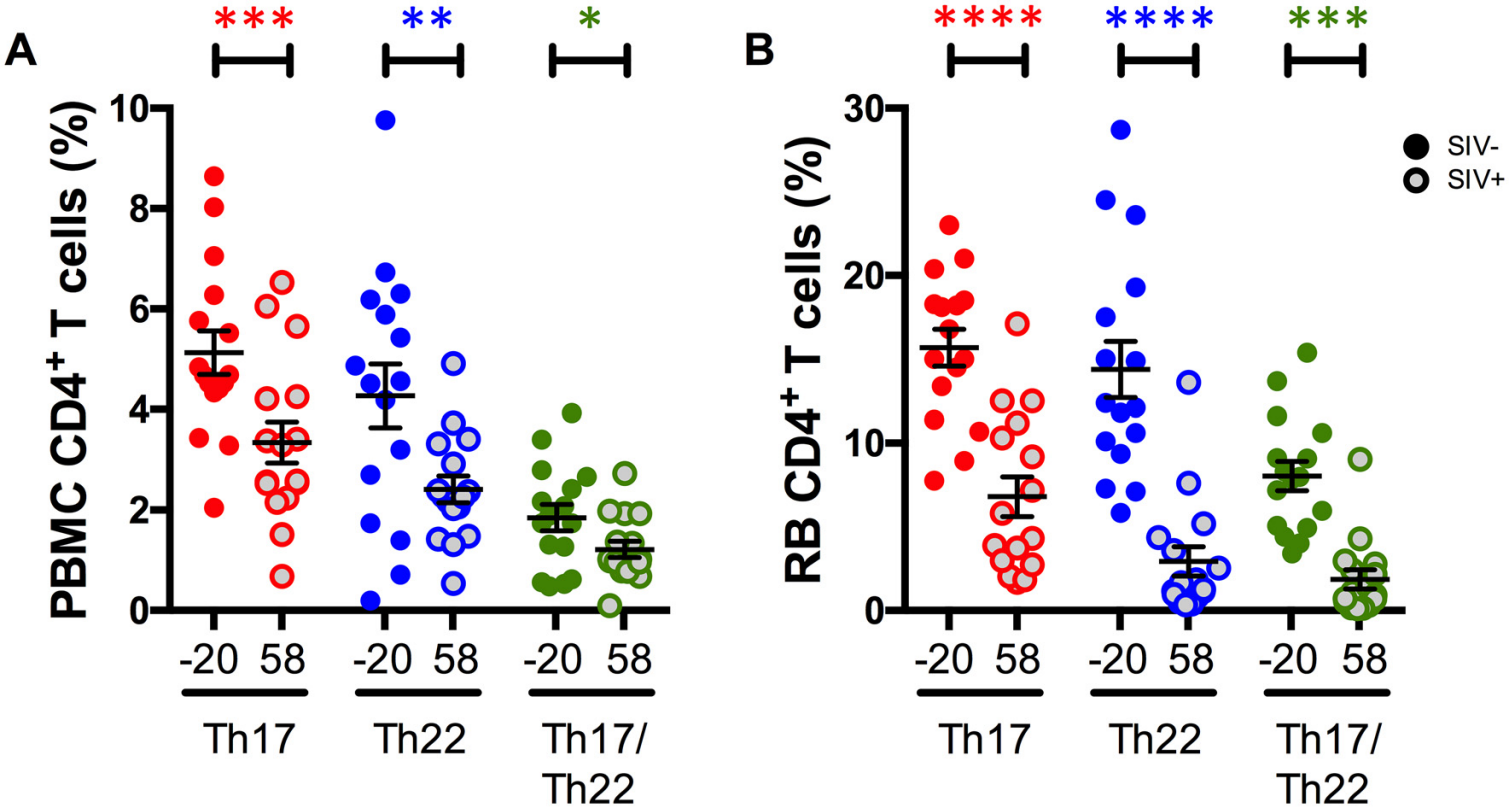
Levels of Th17, Th22, and Th17/Th22 are more drastically depleted by SIV infection in RB than PBMC. Comparison of frequencies (measured as percentages of total CD4^+^ T-cell populations) of circulating **(A)** and colorectal **(B)** Th17 (red), Th22 (blue), and Th17/Th22 (green) cells before (d.-20 p.i.) and after (d. 58 p.i.) SIV infection. Averaged data are presented as means ± SEM.

**Fig 4:**
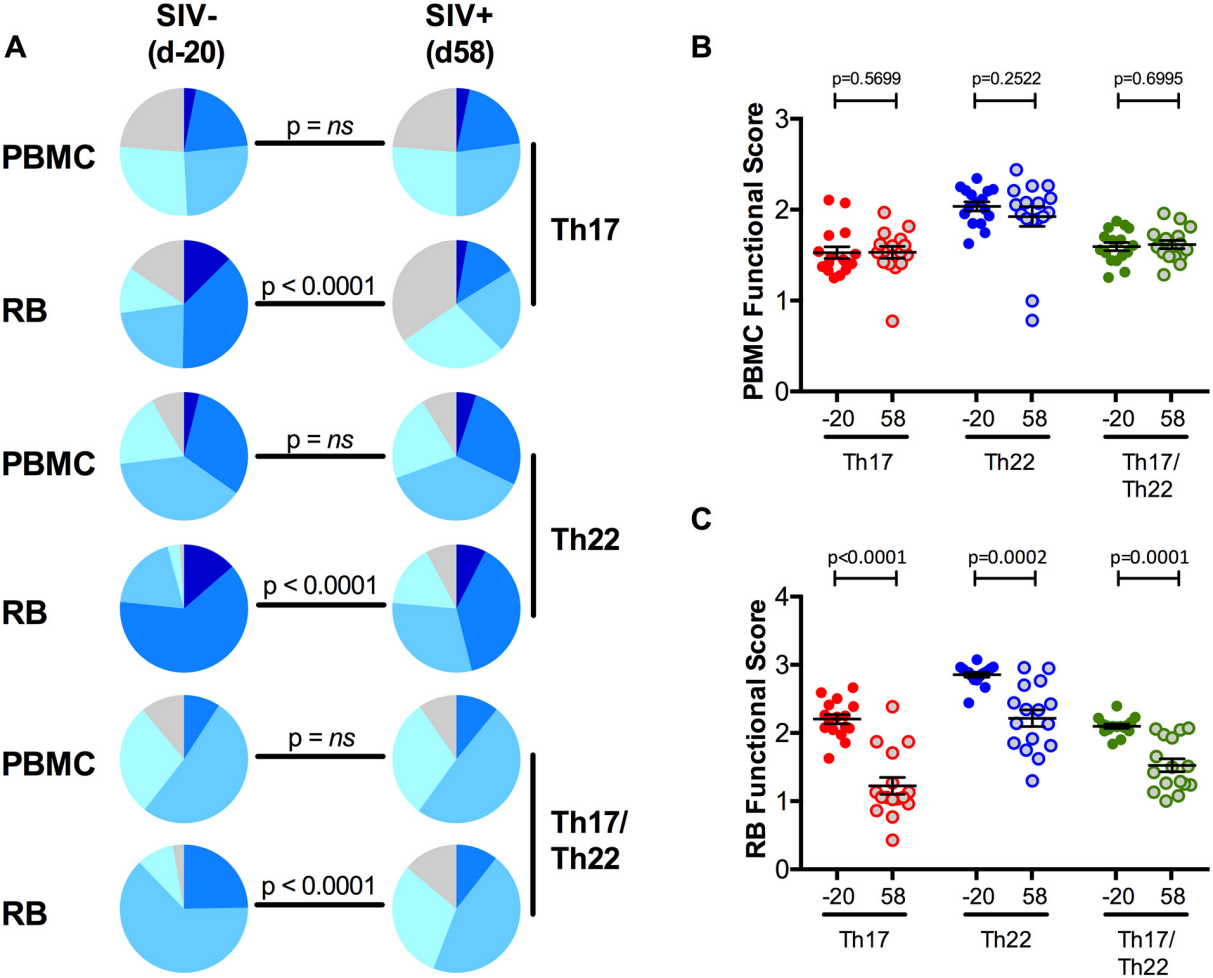
SIV infection severely ablates intestinal IL-17 and IL-22 producing cell function and levels. **(A)** Comparison between PBMC and RB Th17, Th22, and Th17/Th22 cell cytokine profiles at pre-infection (d.-20 p.i.) vs SIV infection (d. 58 p.i.). While all RB cytokine profiles significantly changed after SIV infection, the blood cytokine profiles remained unchanged. **(B)** No changes in functional score in PBMC after SIV infection. **(C)** In all three subsets, colorectal functional scores drastically decreased after SIV infection. Th17 cells marked as red, Th22 cells marked as blue, and Th17/Th22 cells marked as green. Averaged data are presented as means ± SEM.

We then investigated how SIV infection in RMs impacts the frequency and function of IL-17 and IL-22 producing CD4^+^ T-cells. To this aim, the frequencies (Fig 3) and function (Fig 4) of IL-17^+^, IL-22^+^ and IL-17^+^IL-22^+^ CD4^+^ T-cells were compared in the same 16 RMs before (day -20) and post (day 58) SIV infection (p.i.). At day 58 p.i., the means ± S.E were 694,901 ± 244,099 for viral load (copies viral RNA per ml of plasma) and 549 ± 60 for CD4 T cell counts (cells for mm^3^ of blood). In SIV-infected RMs the levels of intestinal IL-17, IL-22, and IL-17/IL-22 producing cells, expressed as fractions of total CD4^+^ T-cells, were all severely depleted by day 58 p.i. (p<0.0001 for all three subsets) (Fig 3B). Significant depletion was also observed in the blood (p = 0.0002 for IL-17^+^; p = 0.0048 for IL-22^+^; and p = 0.0182 for IL-17^+^IL-22^+^; Fig 3A), although its extent was not as substantial as seen in the colorectum.

We then sought to determine if SIV infection also impairs the function of IL-17, IL-22, and IL-17/IL-22 producing CD4^+^ T-cells. The cytokine profiles and functional scores of IL-17, IL-22 or IL-17/IL-22 producing CD4^+^ T-cells remained similar between pre and post SIV infection in blood (Fig 4A and 4B), but were severely altered in the mucosa of SIV-infected animals (Fig 4A and 4C). In particular, at day 58 p.i., we found a significant loss in the proportion of Th17 and Th22 cells co-producing four or five cytokines (p<0.0001), with a concomitant expansion of cells able to produce only their signature cytokine (IL-17 or IL-22), or their signature cytokine plus a single additional cytokine (p<0.0001). A similar loss of function was found in Th17/Th22 CD4^+^ T-cells (p<0.0001). Consistently, the functional score fell from 2.2 ± 0.07 to 1.23 ± 0.12 for Th17 cells (p<0.0001), from 2.86 ± 0.04 to 2.22 ± 0.12 for Th22 cells (p = 0.0002), and from 2.1 ± 0.03 to 1.53 ± 0.09 from Th17/Th22 cells (p = 0.0001), (Fig 4C).

In summary, pathogenic SIV infection in RMs specifically impacts the critical intestinal Th17, Th22, and Th17/Th22 CD4^+^ T-cell compartments both quantitatively and qualitatively, with a severe numeric and functional loss of these cells.

### ART does not fully restore Th17 cell frequencies and functions

**Fig 5:**
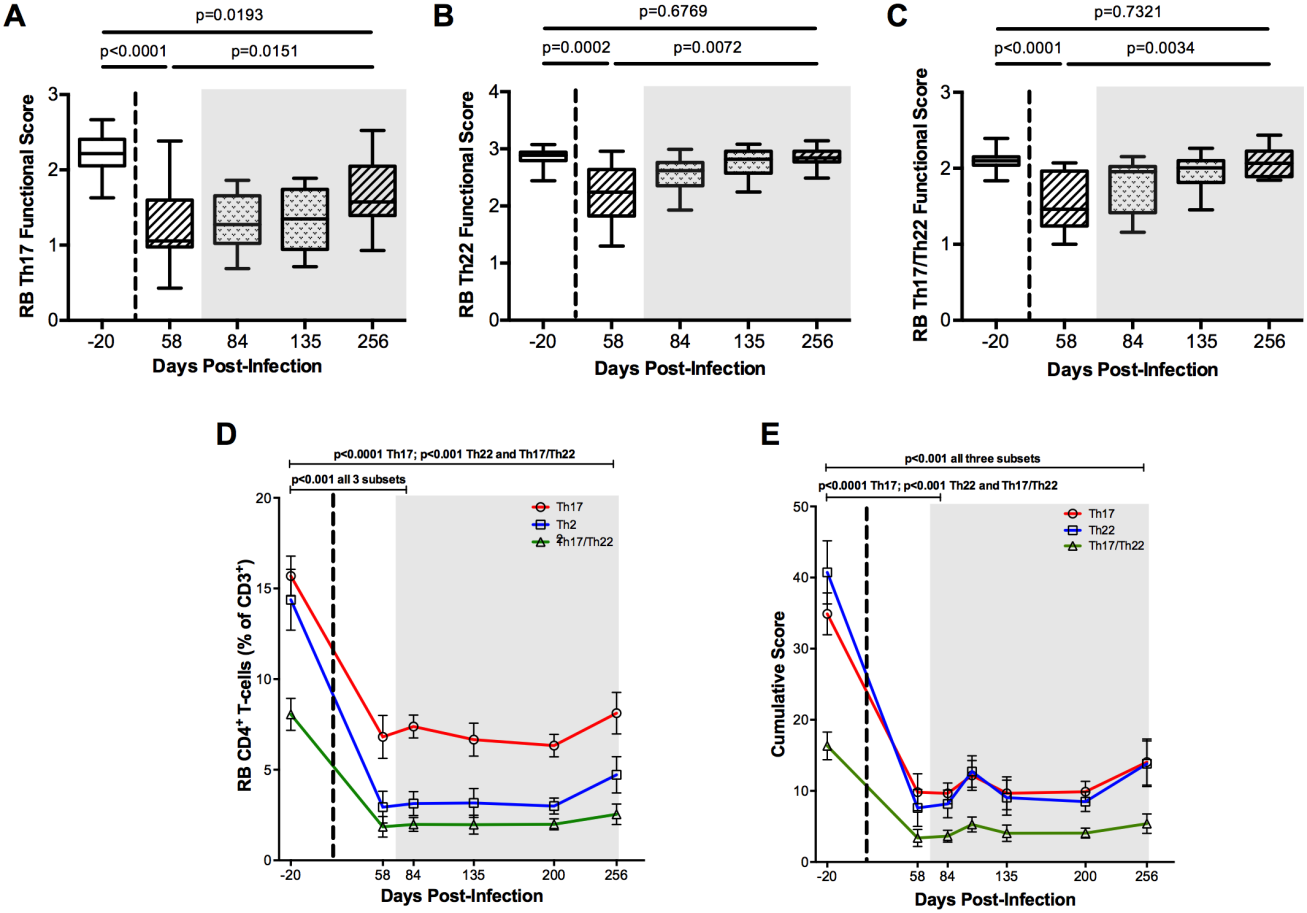
ART treatment is insufficient for full restoration of cell subset levels and function. **(A-C)** Longitudinal functional scores of Th17, Th22, and Th17/Th22 cells in RB at pre-infection (d. -20 p.i.), pre-ART (d. 58 p.i.), and throughout ART (d. 84 p.i. through d. 256 p.i.). Data are presented as box and whisker plots, with the median functional score plotted in between the 25% and 75% quartiles. Dotted line marks time of SIV infection and shaded gray box represents time of ART treatment. ART significantly increased all three subsets’ functional scores, but did not bring Th17 cell function back to pre-infection level. **(D)** Levels of subsets (as percentages of total CD4^+^ T-cell populations) in RB during ART remain significantly lower than pre-infection. **(E)** Longitudinal cumulative subset scores, calculated by multiplying cell frequencies and functional scores, showed the inability of ART to fully restore the levels and function of Th17, Th22, and Th17/Th22 cells. Th17 cells marked as red, Th22 cells marked as blue, and Th17/Th22 cells marked as green. Averaged data are presented as means ± SEM.

We then sought to determine the efficacy of combined antiretroviral therapy (ART) to reconstitute intestinal Th17, Th22, and Th17/Th22 CD4^+^ T-cells. Specifically, number and function of IL-17, IL-22, and IL-17/IL-22 producing CD4^+^ T-cells were longitudinally determined in blood, LN and colorectal mucosa before ART (day 58 p.i.) and at four experimental points during ART (days 84, 135, 200, and 256 p.i., i.e., weeks 3, 10, 20, and 28 on ART), in eight of the original 16 SIV-infected RMs included in this study (RbT12, RGe12, RCb12, RJw12, RPu12, RKg11, RKd12 and RPy8). All eight treated animals showed undetectable (< 60 copies/ml of plasma) levels of viral SIV-RNA starting from 20 weeks on ART (day 200 p.i.). While the lymph node IL-17 producing cells’ function and cytokine profiles were unchanged throughout treatment (S1 Fig), ART was very effective in improving the cytokine profile of the intestinal Th22 cell subset, specifically expanding the proportion of cells co-expressing 3 and 4 cytokines (p = 0.0005, S2 Fig). In fact, the Th22 functional score significantly rose from 2.22 ± 0.12 at pre-ART to 2.85 ± 0.07 at the end of ART treatment (p = 0.0072), thus virtually matching the function level seen before infection (2.86 ± 0.04; p = 0.6769; Fig 5B). Similar results were found for the intestinal Th17/Th22 cells, whose functional scores increased from 1.53 ± 0.09 at pre-ART to 2.08 ± 0.07 at the end of ART (p = 0.0034), matching pre-infection levels (2.08 ± 0.07 vs. 2.09 ± 0.03; p = 0.7321; Fig 5C). In contrast, intestinal Th17 cells were not fully restored by ART. Indeed, although the Th17 cell functional score increased from 1.23 ± 0.12 cytokines at pre-ART to 1.64 ± 0.17 cytokines at the last experimental point on ART (p = 0.0151), it remained significantly lower when compared to that at pre-infection (2.20 ± 0.07 cytokines; p = 0.0193; Fig 5A). We also quantified the levels of intestinal IL-17 and IL-22 producing cells, and here ART’s reconstitution was minimal, with all three subsets’ levels remaining significantly lower p<0.001) than pre-infection levels (Fig 5D). When cell function and subset levels were cumulatively combined, ART’s inability (at least when limited to a 7 month duration as in this study) to fully restore the subsets’ functional and numeric levels was clearly demonstrated (Fig 5E), with cumulative scores at the last experimental point on ART still remarkably lower than those at pre-infection for intestinal Th17, Th22, and Th17/Th22 CD4^+^ T-cells (p<0.001 for all three subsets).

### Level and function of intestinal IL-17 and IL-22 producing CD4^+^ T-cells are rapidly and severely affected after structured ART interruption

**Fig 6:**
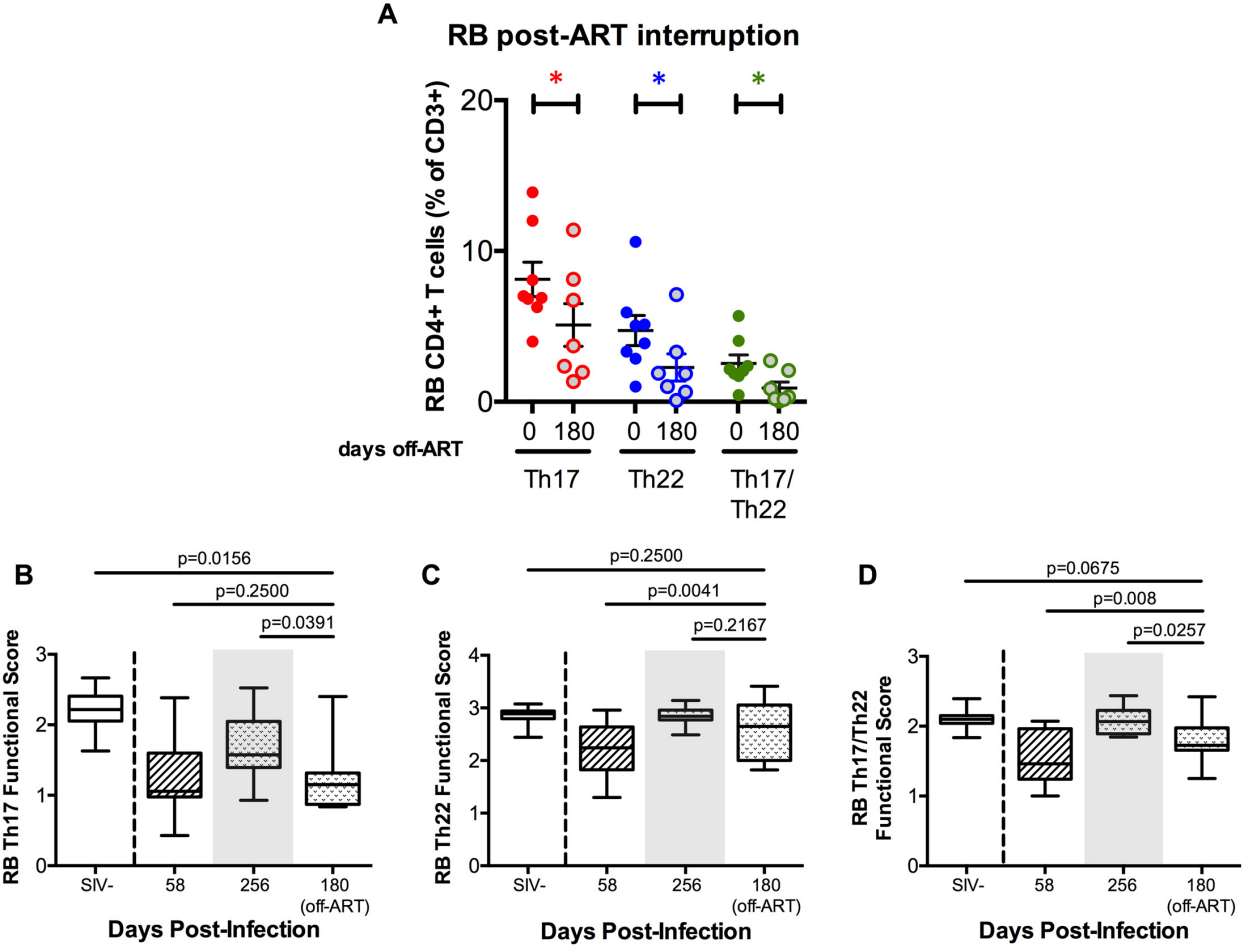
ART interruption severely decreases IL-17 and IL-22 producing cell function and levels. **(A)** Levels of RB Th17 (red), Th22 (blue), and Th17/Th22 (green) cells, measured as percentages of total CD4^+^ T-cell population, significantly decreased following ART interruption (180 days off-ART). Averaged data are presented as means ± SEM. **(B-D**) Longitudinal functional scores of Th17, Th22, and Th17/Th22 cells in RB at pre-infection (d. -20 p.i.), pre-ART (d. 58 p.i.), on-ART treatment (d. 256 p.i.), and after ART interruption (180 days off-ART). ART interruption significantly decreases Th17 and Th17/Th22 cell functional scores from late-ART levels. Dotted line marks time of SIV infection and shaded gray box represents time of ART treatment. Averaged data are presented as box and whisker plots, with the median functional score in between the 25% and 75% quartiles.

We then investigated how the function and levels of IL-17 and IL-22 producing cells were affected with the discontinuation of ART. At day 180 post structured ART interruption, the levels of all three subsets significantly decreased from those seen on ART (p = 0.0416 for Th17; p = 0.0313 for Th22; p = 0.0469 for Th17/Th22; Fig 6A). Importantly, the Th17 cell cytokine profile changed from that observed late on-ART (p<0.0001) and reverted back to the same profile found at pre-ART (p = 0.6600; S2 Fig). Functional score analysis confirmed the drastic fall in Th17 cell function after ART discontinuation, with functional score dropping from 1.67 ± 0.17 to 1.24 ± 0.18 cytokines per cell (p = 0.0391; Fig 6B), and reverting back to pre-ART levels (p = 0.2500; Fig 6B). A similar functional decrease after ART interruption was also observed in the Th17/Th22 cells, whose functional score decreased from 2.08 ± 0.03 (on ART) to 1.79 ± 0.12 (at d180 post ART interruption) (p = 0.0257; Fig 6D). Interestingly, although the cytokine profile changed from on-ART levels, with a reduced fraction of Th22 cells co-producing three cytokines (p<0.0001), the overall functional score for Th22 cells after ART interruption remained unchanged from ART levels (p = 0.2167; Fig 6C). In blood, no significant changes were seen in the cytokine profiles or function after ART discontinuation (S3 Fig).

In summary, and despite its inability for full restoration, ART is necessary for at least maintenance of colorectal Th17, Th22, and Th17/Th22 cell function and levels, as evidenced by the rapid general regression of both upon ART interruption.

### Loss of function of intestinal Th17 and Th22 cells is associated with immune activation and CD4^+^ T-cell loss in SIV-infected RMs

**Fig 7:**
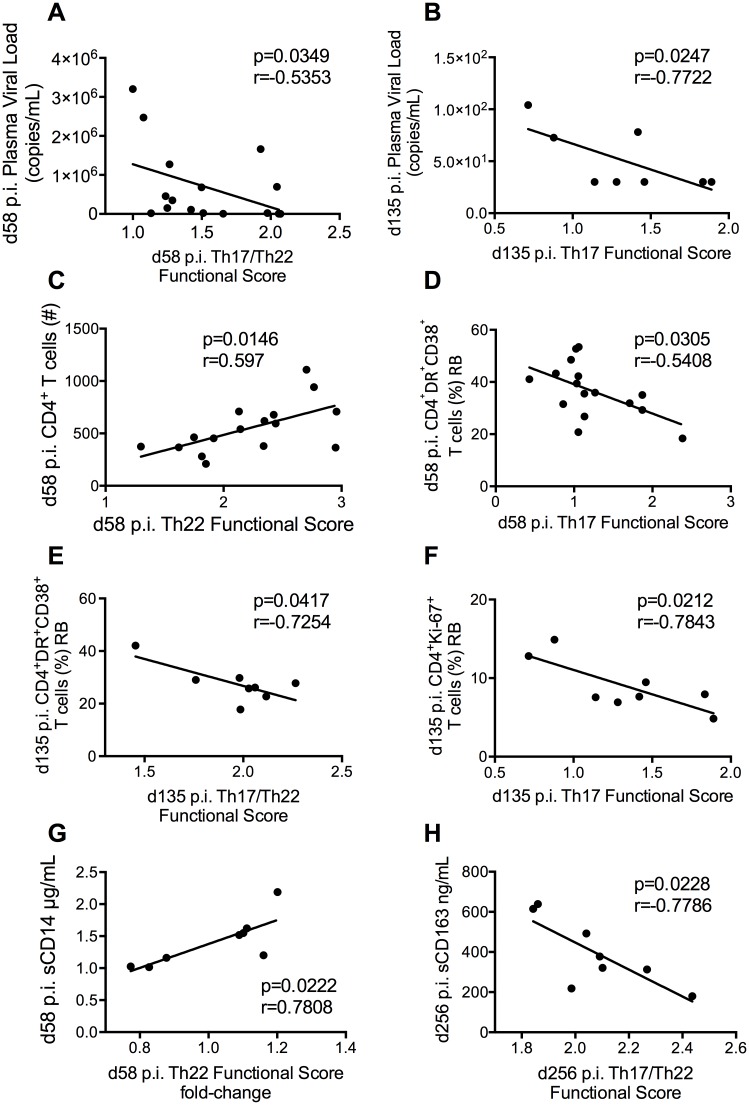
Loss of Th17, Th22, and Th17/Th22 cell function correlates with colorectal immune activation, soluble markers of inflammation, and levels of CD4^+^ T-cells. Correlations between Th17, Th22, and Th17/Th22 cell function and plasma viral loads (copies viral RNA per mL of plasma) **(A,B)**, absolute number of CD4^+^ T-cells **(C)**, levels of activated colorectal (HLA-DR^+^CD38^+^) CD4^+^ T-cells **(D,E)**, the level of proliferating (Ki-67^+^) RB CD4^+^ T-cells **(F)**, as well as soluble markers of inflammation sCD14 and sCD163 **(G, H)**. Pearson product-moment correlation coefficients were measured for all plots except for 7A, which required Spearman’s rank correlation coefficient.

We next determined if the functional impairment (quantified and represented by the functional scores) of the intestinal Th17, Th22 and Th17/Th22 cells associated with the main virologic and immunologic markers of disease progression. We found a negative correlation between Th17/Th22 T-cell function and viral loads at d58 p.i. (p = 0.0349; r = -0.5353; Fig 7A). This same pattern was observed at the intermediate on-ART time point of day 135, in which Th17 (p = 0.0247, r = -0.7722; Fig 7B), Th22 (p = 0.0195, r = -0.7907; S4 Fig), and Th17/Th22 T-cells (p = 0.0063, r = -0.8592; S4 Fig) increased their functional scores while the viral loads decreased, thus suggesting a link between intestinal CD4^+^ T-cell functionality and viral replication. Moreover, absolute CD4^+^ T-cell counts positively correlated with intestinal Th22 cell functional score (p = 0.0146, r = 0.5970) (Fig 7C). To further explore the association between reduced IL-17 producing CD4^+^ T-cell function and disease progression, we also examined levels of colorectal CD4^+^ T-cell proliferation (as measured by Ki-67 expression) and immune activation (as measured by the co-expression of HLA-DR and CD38). At day 58 p.i. (pre-ART), we found a negative correlation between intestinal Th17 cell functional scores and the fraction of HLA-DR^+^CD38^+^ CD4^+^ T-cell levels (p = 0.0305, r = -0.5408, Fig 7D). Similarly, at day 135 p.i., a negative correlation was also seen between Th17/Th22 cell functional scores and the levels of CD4^+^ T-cells co-expressing HLA-DR and CD38 (p = 0.0417, r = -0.7254; Fig 7E). Furthermore, the intestinal Th17 (p = 0.0212, r = -0.7843; Fig 7F), Th22 (p = 0.0285, r = -0.7605; S5 Fig), and Th17/Th22 (p = 0.0353, r = -0.7413; S5 Fig) cell functional scores negatively correlated with the levels of proliferation during ART (day 135 p.i.). We also expanded the analyses to IL-17^+^IFN-γ^+^ CD4^+^ T-cells, as the emergence of this population with a mixed Th17/Th1 phenotype has been proposed to be triggered in pro-inflammatory conditions [47–49]. The frequencies (within the total CD4^+^ T-cell population) of intestinal IL-17^+^IFN-γ^+^ cells significantly decreased upon SIV infection (day 58 p.i.) as compared to pre-infection (day -20 p.i: 3.303 ± 0.3027; day 58 p.i: 1.088 ± 0.3906; mean ± S.E; p = 0.0017), and were significantly increased with ART treatment (day 256 p.i; 1.979 ± 0.7622; mean ± S.E) as compared to day 58 p.i. (p = 0.0156; S6 Fig). Interestingly, while the frequencies of RB IFN-γ^+^ cells associated with levels of RB CD4^+^ DR^+^38^+^ T-cells at the majority of measured time points, including before, throughout, and after ART treatment, IL-17^+^IFN-γ^+^ CD4^+^ T-cells only correlated with levels of RB CD4^+^ DR^+^38^+^ T-cells at day 58 p.i., and never associated with levels of CD8^+^ immune activation or with measures of CD4 and CD8 T-cell proliferation.

Next, we longitudinally examined several soluble markers of immune activation, such as IP-10, sCD14, lipopolysaccharides (LPS), lipopolysaccharide binding protein (LBP), and sCD163, in order to monitor the inflammation status at pre and post-ART initiation. Levels of sCD14, which has been shown to be elevated and to predict mortality in HIV-infected individuals [50], significantly correlated with the functional score fold change between day 256 p.i. and day 58 p.i. in Th22 cells (p = 0.0349, r = -0.7424; Fig 7G) and strongly trended in the Th17/Th22 (p = 0.0679, r = -0.6721) population. In addition, furthering the theory that impaired intestinal IL-17 producing CD4^+^ T-cell functionality is mechanistically linked to immune activation and disease progression, higher functional scores of Th17 cells (p = 0.0465, r = -0.7144; S7 Fig) and Th17/Th22 T-cells (p = 0.0228, r = -0.7786; Fig 7H) during late-ART treatment negatively correlated with levels of sCD163, whose expansion has been linked to faster AIDS progression and residual inflammation [51,52]. We saw similar patterns with IP-10 levels at day 200 p.i., which negatively trended with both Th22 and Th17/Th22 T-cell functional scores, lending further evidence to an association between restored mucosal homeostasis and reduced levels of inflammation. Of note, there were no correlations between intestinal T-cell function and plasma levels of LPS and LBP. Additionally, the same was found with colorectal expression of myeloperoxidase (MPO), a proinflammatory, neutrophil-associated enzyme whose levels are elevated in HIV-infected individuals. Finally, and despite these mucosal associations, intestinal Th17, Th22, and Th17/Th22 T-cell functional scores did not associate with immune activation or disease progression parameters in blood or LN.

Taken together, these results further indicate the critical importance of intestinal IL-17 and IL-22 producing CD4^+^ T-cell function in the pathogenesis of SIV infection, particularly in the gut mucosa.

### IL-17 producing T-cell function and fraction loss correlates with and predicts SIV persistence

**Fig 8:**
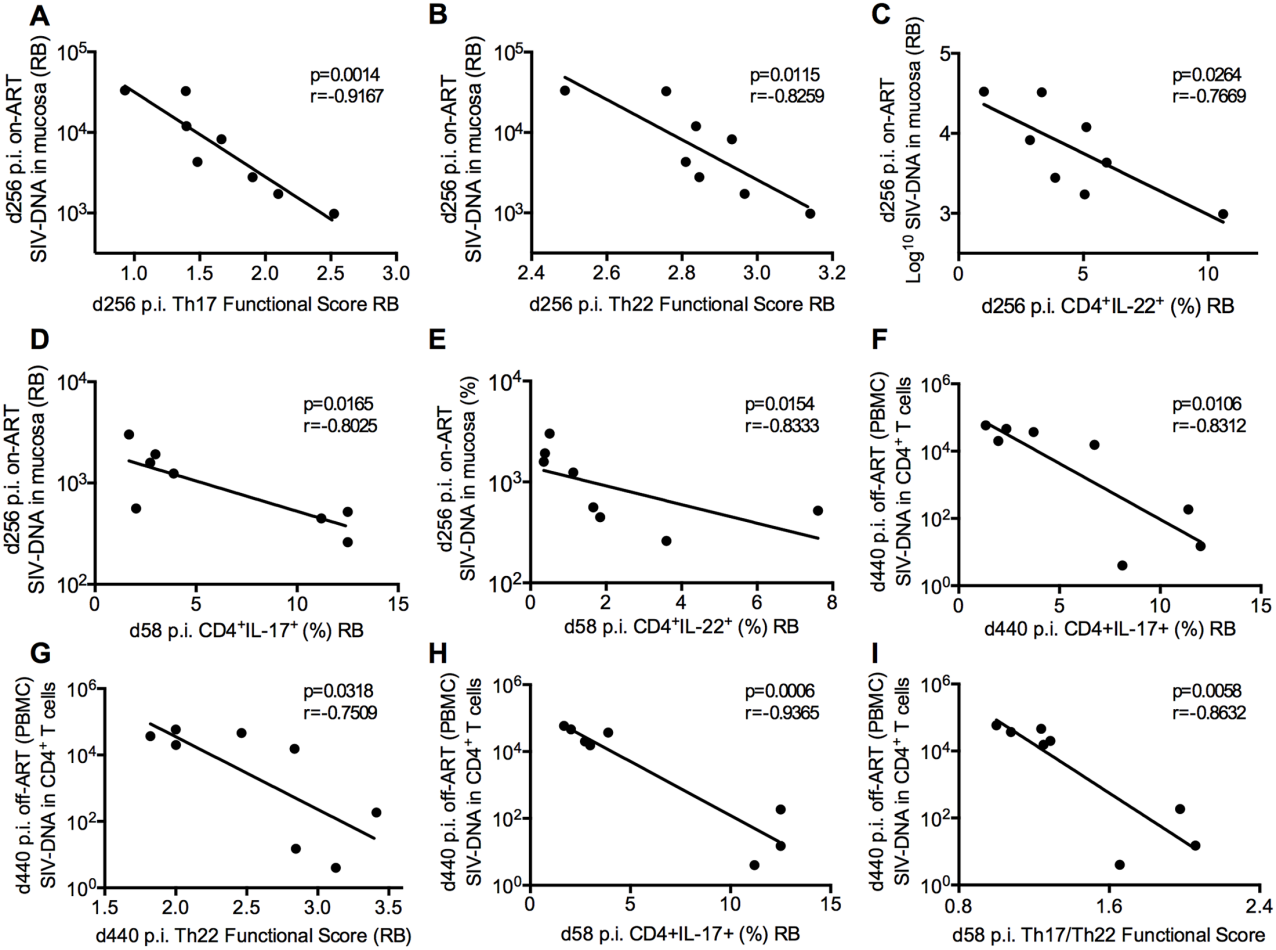
Loss of Th17, Th22, and Th17/Th22 cell function and levels are correlates and predictors of SIV persistence. Correlations between colorectal SIV-DNA content at late-ART (d. 256 p.i.) and IL-17 and IL-22 producing cell function and levels at both ART (**A-C**) and pre-ART infection (d. 58 p.i). **(D,E)**. Levels and function of the three subsets at pre-ART and after ART interruption additionally correlated with levels of blood DNA levels after ART interruption (d. 440 p.i.) **(F-I)**. Pearson product-moment correlation coefficients were measured for all plots except for 8E, which required Spearman’s rank correlation coefficient.

We then investigated whether, by impacting residual activation and inflammation, the Th17, Th22, and Th17/Th22 cell number and function loss associates with SIV persistence on ART. To address this question, we measured at various experimental points the amount of total SIV-DNA in the colorectum and in CD4^+^ T-cells purified from blood. At the last on-ART time point (day 256 p.i.), increasing Th17 (p = 0.0014, r = -0.9167) and Th22 cell (p = 0.0115, r = -0.8259) function, as well as Th22 cell levels (p = 0.0264, r = -0.7669), strongly correlated with lower levels of SIV-DNA in the colorectum (copies per 10^8^ cells equivalent) (Fig 8A–8C). Of note, these associations between Th17 and Th22 cells and SIV-DNA content were independent from and even stronger after controlling for plasma viremia pre-ART (S1 Table). Additionally, this mucosal SIV-DNA content at the latest time point on ART correlated with the fractions of Th17 (p = 0.0165, r = -0.8025) and Th22 (p = 0.0154, r = -0.8333) cells at pre-ART, thus suggesting that IL-17 and IL-22 producing T-cell numbers before treatment can also predict the level of intestinal cell-associated SIV-DNA that will persist during ART (Fig 8D and 8E).

The suggested link between loss of IL-17 and IL-22 producing T-cell function and levels and SIV persistence is furthered by SIV-DNA data from after ART-interruption. Six months after ART had been interrupted (day 440 p.i.), the fraction of Th17 cells (p = 0.0106, r = -0.8312) and Th22 cell function (p = 0.0318, r = -0.7509) both correlated with blood SIV-DNA content (copies per 10^6^ CD4^+^ T-cells) (Fig 8F and 8G). Further, blood SIV-DNA content at six months after ART interruption correlated with Th17 cell levels (p = 0.0006, r = -0.9365) and Th17/Th22 cell function (p = 0.0058, r = -.08632) before ART initiation (day 58 p.i.) (Fig 8H and 8I). Longitudinal values for plasma viremia and blood CD4^+^ T-cells SIV-DNA contents are shown in S8 Fig for the eight animals that underwent ART interruption.

Collectively, these results indicate that animals with higher function and numbers of IL-17, IL-22, and IL-17/IL-22 producing cells before and during ART treatment exhibit lower SIV-DNA content throughout and after ART treatment, thus suggesting Th17, Th22, and Th17/Th22 cell numbers and function as critical regulators of SIV persistence.

## Discussion

A severe loss of intestinal CD4^+^ T cells [1–4], which preferentially involves CD4^+^ T-cell subsets with anti-microbial properties such as Th17 and Th22 cells [29,30,32–34,53], has been proposed as one of the most critical factors contributing to the breakdown of mucosal epithelial integrity during chronic HIV and SIV infections. This loss of mucosal integrity is considered a key contributor to the establishment and persistence of high levels of chronic immune activation in HIV infection [34,36,54]. While several studies have convincingly shown a preferential loss of intestinal IL-17 and IL-22 producing CD4^+^ T-cells in HIV and SIV infection, it is unclear how exactly the functions of these cells are perturbed during infection. Furthermore, the extent to which the number and function of IL-17 and IL-22 producing CD4^+^ T-cells are recovered during highly effective ART therapy is still unclear. The only limited insight to these important questions comes from a few HIV studies that documented the loss of IL-17 producing cell function during progressive infection and the challenge in reversing this defect, with Th17 cell function fully recovered only after extremely long-term ART, particularly that which was started very early after infection [45,46].

To provide better insights on these important questions, we used the HIV model of SIV infection in RMs to extensively investigate the effects of SIV infection, ART treatment, as well as ART interruption on the number and functional ability of IL-17 and IL-22 producing CD4^+^ T-cells to simultaneously produce multiple effector cytokines such as IL-17, IL-22, TNFα, IFNγ, and IL-2. While several recent cross-sectional studies have linked decreased Th17 cell function to HIV infection and increased immune activation, none have attempted to follow changing functional dynamics longitudinally. A key advantage of our study was the possibility to follow the same animals longitudinally throughout pre and post SIV infection, ART treatment, as well as after ART interruption, which is virtually impossible in human studies due to ethical, demographic, and time constraints. Another strength in our investigation was that Th22 and Th17/Th22 cell function were also simultaneously examined, as several recent studies have indicated that Th17 cells are not the only subset of CD4^+^ T-cells preferentially lost from the intestines of HIV and SIV infected individuals. In addition, this quantification of IL-17 and IL-22 producing CD4^+^ T-cell levels and function was simultaneously investigated in blood, colorectum, as well as in the lymph nodes. This provided us with the unique opportunity to investigate the extent of association between numerous immunologic and virologic correlates of disease progression, as well as SIV persistence and total DNA content, and IL-17 producing cell function. To the best of our knowledge, these longitudinal, in-depth studies on the number and functional abilities of IL-17 and IL-22 producing CD4^+^ T-cells have never been performed in HIV-infected humans or SIV-infected RMs.

We found that intestinal, but not blood or lymph node, IL-17, IL-22, and IL-17/IL-22 producing CD4^+^ T-cells are polyfunctional in SIV-uninfected RMs, with the large majority of the cell types co-producing four or five cytokines. However, SIV infection induced a numeric loss and a severe dysfunction of these intestinal CD4^+^ T-cells and caused the average numbers of cytokines produced per each cell to drastically drop, while leaving blood and lymph node cytokine expression profiles unchanged, thus supporting the critical importance of intestinal IL-17 and IL-22 producing CD4^+^ T-cells in maintaining mucosal homeostasis during HIV and SIV infection.

We examined how ART treatment at different time points and after its interruption affected cell cytokine expression dynamics and functional levels. Our results give strong evidence that ART partially restores and maintains colorectal Th17, Th22, and Th17/Th22 CD4^+^ T-cell polyfunctionality, especially evidenced by severe decreases in level and function observed when ART was interrupted. However, it is clear that ART alone, at least when used for 7 months as in our study, is not sufficient to bring cell function and numbers back to pre-infection levels, as all examined subsets by the end of ART showed significantly reduced cumulative levels; this was particularly noticeable in the case of the Th17 cells.

Importantly, we also found that the loss of intestinal IL-17 and IL-22 producing CD4^+^ T-cell function associated with the extent of chronic immune activation, pre-ART viral loads, as well as lower CD4^+^ T-cell counts before and during ART treatment. Furthermore, levels of gut T-cell activation and proliferation were associated with loss of function from all three (IL-17, IL-22, and IL-17/IL-22 producing) intestinal cell subsets. In addition, cell function negatively correlated with levels of sCD163 and sCD14, two soluble markers of inflammation, whose levels have been shown to increase during HIV and SIV infection and are linked to microbial translocation, macrophage activation, disease progression, and mortality in ART-treated HIV-infected individuals [50–52]. Remarkably, this loss of function and cell frequency also consistently correlated with higher SIV-DNA content in the colorectum and blood throughout and after ART, thus suggesting IL-17 and IL-22 producing CD4^+^ T-cell numbers and function as regulators and potential predictors of SIV persistence.

While the design of our study has allowed for novel findings and a longitudinal investigation into the nature of IL-17 and IL-22 producing cell frequency and function in the context of SIV infection, there exist several confining factors. Our study utilized a 7 month ART duration, largely dictated by the practicality of using a nonhuman primate model. Based on our data, we cannot exclude that a more prolonged therapy and/or earlier initiation would have resulted in a more significant restoration of Th17 cell number and function. A recent study in HIV-infected humans showed Th17 cell function, after having been drastically ablated after infection, was eventually fully restored to pre-infection levels, but only after very prolonged ART (median of 13 years) [44]. Additionally, two other studies showed that intestinal Th17 cell function was preserved when ART was initiated very early during acute infection [45,46]. In addition, since our analyses during the natural history of SIV-infection were performed in the early chronic phase of infection (day 58 p.i.), we cannot determine how the function of IL-17 and IL-22 producing CD4^+^ T-cells are affected in the early acute infection. Further studies focusing on the first days after SIV infection are needed to address this important point.

In conclusion, we demonstrated that the polyfunctionality specific to intestinal IL-17 and IL-22 producing CD4^+^ T-cells is severely compromised upon SIV infection. ART does not fully restore the function and levels in these mucosal Th17, Th22, and Th17/Th22 T-cells, yet is necessary for at least partial functional maintenance, as evidenced by the ablative effects of ART interruption. Importantly, this loss of IL-17 and IL-22 producing cell function and frequency associates with and predicts immune activation and disease progression in RMs, as well as SIV persistence in colorectum and blood. As such, our data suggests that therapies able to preserve and/or regenerate the functions of these intestinal CD4^+^ T-cells subsets central for mucosal immunity should be included in the therapeutic regimen necessary for achieving HIV remission.

## Materials and Methods

### Ethics statement

All animal experimentations were conducted following guidelines established by the Animal Welfare Act and the NIH for housing and care of laboratory animals and performed in accordance with Institutional regulations after review and approval by the Institutional Animal Care and Usage Committees (IACUC; protocol #2001973) at the Yerkes National Primate Research Center (YNPRC). Appropriate procedures were performed to ensure that potential distress, pain, discomfort and/or injury was limited to that unavoidable in the conduct of the research plan. All the blood and tissue collections were obtained from RMs housed at the Yerkes National Primate Research Center, which is accredited by American Association of Accreditation of Laboratory Animal Care. The sedative Ketamine (10 mg/kg) and/or Telazol (4 mg/kg) were applied as necessary for blood and tissue collections and analgesics were used when determined appropriate by veterinary medical staff. RMs were fed standard monkey chow (Jumbo Monkey Diet 5037, Purina Mills, St Louis, MO) twice daily. Consumption is monitored and adjustments are made as necessary depending on sex, age, and weight so that animals get enough food with minimum waste. SIV-infected RMs are singly caged but have visual, auditory, and olfactory contact with at least one social partner, permitting the expression of non-contact social behavior. The YNPRC enrichment plan employs several general categories of enrichment. Animals have access to more than one category of enrichment. IACUC proposals include a written scientific justification for any exclusions from some or all parts of the plan. Research-related exemptions are reviewed no less than annually. Clinically justified exemptions are reviewed more frequently by the attending veterinarian.

### Animals, SIV-infection, and ART regimen

Sixteen RMs, all housed at the Yerkes National Primate Research Center, Atlanta, GA, were included in the study. All animals were *Mamu*-B*08 and B*17 negative, while eight of them were *Mamu*-A*01 positive (RLm12, RBt12, RJp11, RCb12, RVt10, RKg11, ROc10 and RPy8). The 16 RM were in average 9.1 ± 0.6 years old and weighed 7 ± 0.28 kg. All 16 animals were infected intravenously (i.v.) with 300 TCID_50_ SIVmac_239_ (day 0). Starting at day 58 p.i., all animals were treated with a five-drug ART regimen comprising of two reverse transcriptase (RT) inhibitors (PMPA 20 mg/Kg and FTC 30 mg/Kg), one integrase inhibitor (raltegravir 100 mg/bid), and one protease inhibitor (darunavir 375 mg/bid with ritonavir 50 mg/bid as a boosting supplementation) for seven months. Animal ROc10 was euthanized at d140 p.i. due to post-surgery (lymph node biopsy) related complications. At day 270 p.i., ART was interrupted and all animals were monitored for an additional eight months. Peripheral blood (PB), colorectal mucosa (RB) and lymph node (LN) biopsies were collected at numerous experimental points throughout all the study (Fig 1a).

### Sample collection and processing

Collections and processing of PB, RB, and LN were performed as previously described [29–31,55,56]. All samples were processed, fixed (1% paraformaldehyde), and analyzed within 24 hours of collection.

### Flow cytometric analysis

Fourteen-parameter flow cytometric analysis was performed on PB-, LN- and RB-derived cells. Predetermined optimal concentrations were used of the following antibodies: anti-CD3-APC-Cy7 (clone SP34-2), anti-Ki-67-Alexa700 (clone B56), anti-IFN-γ-PE-Cy7 (clone B27), anti-CD8-PE-CF-594 (clone RPA-T8), anti-TNFα-Alexa700 (clone MAb11), (all from BD Pharmingen); anti-IL-17-Alexa Fluor488 (clone eBio64DEC17), anti-IL-22-APC (clone IL22JOP) (all from eBioscience); anti-CD4-BV421 (clone OKT4), anti-IL-2-BV605 (clone MQ1-17H12), (all from Biolegend); anti-CD8-Qdot705 (clone 3B5) and Aqua Live/Dead amine dye-AmCyan (all from Invitrogen). Flow cytometric acquisition was performed on at least 100,000 CD3^+^ T cells on an LSRII cytometer driven by the FACS DiVa software. Analysis of the acquired data was performed using FlowJo software (TreeStar).

### Intracellular cytokine staining

Levels of Th17 and Th22 cells were determined as the percentage of CD4^+^ T-cells that produce IL-17 and IL-22 following *in vitro* stimulation with PMA & Ionomycin [31]. PBMC, LN and RB derived cells, isolated as described above, were resuspended to 3 × 10^6^ cells/ml in complete RPMI 1640 medium. Cells were then incubated for 4 h at 37°C in medium containing PMA, A23187, and Golgi Stop. Following incubation, the cells were washed and stained with surface markers for 30 minutes in the dark at room temperature followed by fixation and permeabilization. After permeabilization, cells were washed and stained intracellularly with the antibodies for the cytokines of interest for 1 hour in the dark at room temperature. Following staining, cells were washed, fixed in PBS containing 1% paraformaldehyde, and acquired on an LSRII cytometer.

### Plasma viral load

Plasma SIV viral loads were determined by standard quantitative RT-PCR as previously described (limit of detection 60 copies/ml) [57].

### SIV-DNA quantification in blood CD4^+^ T-cells and colorectum

Quantitative assessment of cell-associated total SIV-DNA within circulating CD4+ T-cells at days 58 p.i., 105 and 256 on-ART, and day 180 off-ART was performed using a modified version of a recently published quantitative nested PCR assay for cell-associated total HIV-DNA [58]. In a first round of PCR, total SIV DNA was amplified with two primers that anneal within conserved region of the LTR 5’ (SIV-LF1) and at the junction with Gag gene (SIV-R1). The forward primer SIV-LF1 is extended with a lambda phage-specific heel sequence at 5’ end of the oligonucleotide. Primers targeting CD3 gene (HCD3OUT 5’ and HCD3OUT 3’) were also added to quantify the exact number of cells in the initial samples. Gag-LTR sequence were amplified from 15 μL of lysate in a 50 μL reaction mixture comprising 1X Taq Buffer, MgCl2, dNTP, SIV- 38 LF1, SIV-R1 and Taq polymerase. The first round PCR cycle conditions were as follows: a denaturation step of 8 min at 95°C and then 16 cycles of amplification (95°C for 1min, 62°C for 40 sec, 72°C for 1 min), followed by an elongation step at 72°C for 15 min. In a second round of PCR, the lambda T-specific primer (Lambda T) and the LTR primer (SIV-R2), were used to amplified SIV sequences obtained from the first amplification. Primers targeting CD3 were also used in another second round PCR. Nested PCR was performed on 1/10 of the first round PCR product in a mixture comprising 1x Rotor Gene Master mix, Lambda T primer, SIV-R2 primers and SIVprobe. For CD3 amplification, nested PCR was performed in a mixture comprising 1X Rotor Gene Master Mix, HCD3IN 5’ and MamuCD3IN 3’ and MamuCD3probe. The cycling was performed on the Rotorgene (Qiagen) as follow: a denaturation step (95°C for 4 min), followed by 40 cycles of amplification (95°C for 3 sec, 60°C for 10 sec). The copy number of total SIV DNA was calculated by using a standard curve as a reference. This standard curve consisted in serial dilution of the 3D8 cell lysates (carrying one integrated copy of SIV genome per cell) [59].

Quantitative assessments of SIV-DNA in colorectal tissue at days 50 and 200 on-ART were determined by the quantitative hybrid real-time/digital RT–PCR and PCR assays, as previously described [60]. For each sample, 12 replicate reactions were run with a nominal single copy sensitivity. The clinical sensitivity (based on the number of cells assessed) in our samples was as low as 1 copy/850,000 cells.

### Statistical analysis

Based on sample distribution (normal or non-normal), T-tests or Mann Whitney tests were used to compare the differences of each parameter between two different groups. Statistical tests were two-sided. Pearson product-moment correlation coefficients were utilized to estimate linear associations for normally distributed data and Spearman rank correlation coefficients were used for skewed and other non-normal distributions. A P value ≤ 0.05 was considered statistically significant. The mean ± SEM were used for descriptive statistics for each parameter. All linear regression and correlation analyses were performed using Prism version (5) software (GraphPad). IL-17 and IL-22 producing cell polyfunctionality was obtained by FlowJo Boolean gating analysis. SPICE software version 5.33 (National Institute of Allergy and Infection Diseases/National Institutes of Health) was utilized to perform Th17 and Th22 ployfunctionality analysis with the Wilcoxon signed rank test. Functional scores were calculated based on the mean numbers of proinflammatory cytokines produced by each Th17, Th22, or Th17/Th22 cell (specifically calculated by multiplying the fraction of cells in each pie slice by the number of cytokines it represented, and then summing together the resulting values and dividing by 100).

## Supporting Information

S1 Fig Lymph node Th17 and Th22 cell function unchanged throughout ART treatment. Longitudinal assessment of LN Th17 and Th22 cytokine profiles **(A)** and functional score **(B)** at SIV infection (d. 58 p.i.), early ART treatment (d. 84 p.i.), and late ART treatment (d. 256 p.i.). Both cytokine profiles and functional score remained statistically unchanged during ART. Dotted line marks time of SIV infection and shaded gray box represents time of ART treatment. Averaged data are presented as box and whisker plots, with the median functional score in between the 25% and 75% quartiles.
(TIFF)

S2 Fig Intestinal Th22 and Th17 cell cytokine profiles change throughout and after ART. Longitudinal assessment of intestinal Th22 **(A)** and Th17 **(B)** cytokine profiles during chronic SIV infection (d. 58 p.i.), late ART treatment (d. 256 p.i.) and at six months after ART interruption (d. 440 p.i.). Cytokine profiles were generated for each cell population by SPICE program v. 5.33, and were calculated by Flowjo Boolean gating.
(TIFF)

S3 Fig Blood Th17, Th22 and Th17/Th22 cell function and cytokine profiles remain unchanged after ART interruption. Comparison of blood Th17, Th22 and Th17/Th22 functional score **(A)** and cytokine profiles **(B)** between pre (d. 256 p.i.) and post-ART interruption (d. 440 p.i.). Both cytokine profiles and functional score remained statistically unchanged before and after ART discontinuation. Shaded gray box represents time of ART treatment. Averaged data are presented as box and whisker plots, with the median functional score in between the 25% and 75% quartiles. Cytokine profiles were generated for each cell population by SPICE program v. 5.33, and were calculated by Flowjo Boolean gating.
(TIFF)

S4 Fig Intestinal Th22 and Th17/Th22 cell function associates with mid-ART plasma viral loads. Intestinal Th22 **(A)** and Th17/Th22 **(B)** functional scores at d135 p.i. inversely correlate with plasma viral load levels at the same experimental point (d. 135 p.i.).
(TIFF)

S5 Fig Intestinal Th22 and Th17/Th22 cell function negatively associates with cell proliferation at d135 p.i. Intestinal Th22 **(A)** and Th17/Th22 **(B)** functional scores at d135 p.i. negatively correlate with intestinal CD4^+^ T cell proliferation levels (Ki-67^+^).
(TIFF)

S6 Fig Longitudinal levels of intestinal IL-17^+^IFN-γ^+^ CD4^+^ T cells during SIV infection and ART treatment. Intestinal IL-17^+^IFN-γ^+^ CD4^+^ T cells are significantly depleted during chronic SIV infection and not fully restored during the 7 months of ART treatment. Dotted line marks time of SIV infection and shaded gray box represents time of ART treatment. Averaged data are presented as means with SD.
(TIFF)

S7 Fig Intestinal Th17/Th22 and Th17 cell function associates with late-ART levels of sCD163. Higher functional scores of Th17 cells at d256 p.i. negatively correlated with levels of sCD163 at the same experimental point.
(TIFF)

S8 Fig Viral rebound profiles after structured ART interruption. **(A)** Plasma levels of SIVmac239 RNA, expressed as copies/ml and **(B)** peripheral blood SIVmac239 DNA content, expressed as copies/1,000,000 CD4^+^ T-cells, are shown in 8 RMs that underwent structured ART interruption.
(TIFF)

S1 Table Th17 and Th22 functional scores associate with mucosal SIV-DNA content independently from pre-ART viral load. The relationship between intestinal SIV-DNA levels and Th17 and Th22 cell function at d. 256 p.i. was conducted with adjustment for pre-ART (d.58 p.i.) plasma SIVmac239 levels. Adjusted linear regressions models for both subsets were run with the sample size of 8 animals.
(DOCX)

## References

[1] Veazey RS, DeMaria M, Chalifoux LV, Shvetz DE, Pauley DR, et al (1998) Gastrointestinal tract as a major site of CD4+ T cell depletion and viral replication in SIV infection. Science 280: 427–431. 9545219 10.1126/science.280.5362.427

[2] Estes JD, Harris LD, Klatt NR, Tabb B, Pittaluga S, et al (2010) Damaged intestinal epithelial integrity linked to microbial translocation in pathogenic simian immunodeficiency virus infections. PLoS pathogens 6: e1001052 10.1371/journal.ppat.1001052 20808901 PMC2924359

[3] Mehandru S, Poles MA, Tenner-Racz K, Horowitz A, Hurley A, et al (2004) Primary HIV-1 infection is associated with preferential depletion of CD4+ T lymphocytes from effector sites in the gastrointestinal tract. The Journal of experimental medicine 200: 761–770. 15365095 10.1084/jem.20041196 PMC2211967

[4] Brenchley JM, Schacker TW, Ruff LE, Price DA, Taylor JH, et al (2004) CD4+ T cell depletion during all stages of HIV disease occurs predominantly in the gastrointestinal tract. The Journal of experimental medicine 200: 749–759. 15365096 10.1084/jem.20040874 PMC2211962

[5] Schmitt N, Ueno H (2015) Regulation of human helper T cell subset differentiation by cytokines. Current opinion in immunology 34: 130–136. 10.1016/j.coi.2015.03.007 25879814 PMC4465198

[6] Wilson CB, Rowell E, Sekimata M (2009) Epigenetic control of T-helper-cell differentiation. Nature reviews Immunology 9: 91–105. 10.1038/nri2487 19151746

[7] Korn T, Bettelli E, Oukka M, Kuchroo VK (2009) IL-17 and Th17 Cells. Annual review of immunology 27: 485–517. 10.1146/annurev.immunol.021908.132710 19132915

[8] Bettelli E, Korn T, Kuchroo VK (2007) Th17: the third member of the effector T cell trilogy. Current opinion in immunology 19: 652–657. 17766098 10.1016/j.coi.2007.07.020 PMC2288775

[9] Korn T, Oukka M, Kuchroo V, Bettelli E (2007) Th17 cells: effector T cells with inflammatory properties. Seminars in immunology 19: 362–371. 18035554 10.1016/j.smim.2007.10.007 PMC2839934

[10] Chen Z, O'Shea JJ (2008) Th17 cells: a new fate for differentiating helper T cells. Immunol Res 41: 87–102. 10.1007/s12026-007-8014-9 18172584

[11] Acosta-Rodriguez EV, Rivino L, Geginat J, Jarrossay D, Gattorno M, et al (2007) Surface phenotype and antigenic specificity of human interleukin 17-producing T helper memory cells. Nature immunology 8: 639–646. 17486092 10.1038/ni1467

[12] Lee JS, Cua DJ (2015) IL-26 AMPs up the TH17 arsenal. Nature immunology 16: 897–898. 10.1038/ni.3256 26287587

[13] Meller S, Di Domizio J, Voo KS, Friedrich HC, Chamilos G, et al (2015) TH17 cells promote microbial killing and innate immune sensing of DNA via interleukin 26. Nature immunology 16: 970–979. 10.1038/ni.3211 26168081 PMC4776746

[14] Duhen T, Geiger R, Jarrossay D, Lanzavecchia A, Sallusto F (2009) Production of interleukin 22 but not interleukin 17 by a subset of human skin-homing memory T cells. Nature immunology 10: 857–863. 10.1038/ni.1767 19578369

[15] Trifari S, Kaplan CD, Tran EH, Crellin NK, Spits H (2009) Identification of a human helper T cell population that has abundant production of interleukin 22 and is distinct from T(H)-17, T(H)1 and T(H)2 cells. Nature immunology 10: 864–871. 10.1038/ni.1770 19578368

[16] Ramirez JM, Brembilla NC, Sorg O, Chicheportiche R, Matthes T, et al (2010) Activation of the aryl hydrocarbon receptor reveals distinct requirements for IL-22 and IL-17 production by human T helper cells. European journal of immunology 40: 2450–2459. 10.1002/eji.201040461 20706985

[17] Leung JM, Davenport M, Wolff MJ, Wiens KE, Abidi WM, et al (2014) IL-22-producing CD4+ cells are depleted in actively inflamed colitis tissue. Mucosal immunology 7: 124–133. 10.1038/mi.2013.31 23695510 PMC3870042

[18] Pickert G, Neufert C, Leppkes M, Zheng Y, Wittkopf N, et al (2009) STAT3 links IL-22 signaling in intestinal epithelial cells to mucosal wound healing. The Journal of experimental medicine 206: 1465–1472. 10.1084/jem.20082683 19564350 PMC2715097

[19] Sugimoto K, Ogawa A, Mizoguchi E, Shimomura Y, Andoh A, et al (2008) IL-22 ameliorates intestinal inflammation in a mouse model of ulcerative colitis. The Journal of clinical investigation 118: 534–544. 10.1172/JCI33194 18172556 PMC2157567

[20] Wolk K, Kunz S, Witte E, Friedrich M, Asadullah K, et al (2004) IL-22 increases the innate immunity of tissues. Immunity 21: 241–254. 15308104 10.1016/j.immuni.2004.07.007

[21] Zheng Y, Valdez PA, Danilenko DM, Hu Y, Sa SM, et al (2008) Interleukin-22 mediates early host defense against attaching and effacing bacterial pathogens. Nat Med 14: 282–289. 10.1038/nm1720 18264109

[22] O'Connor W Jr., Zenewicz LA, Flavell RA (2010) The dual nature of T(H)17 cells: shifting the focus to function. Nature immunology 11: 471–476. 10.1038/ni.1882 20485275

[23] Ota N, Wong K, Valdez PA, Zheng Y, Crellin NK, et al (2011) IL-22 bridges the lymphotoxin pathway with the maintenance of colonic lymphoid structures during infection with Citrobacter rodentium. Nature immunology 12: 941–948. 10.1038/ni.2089 21874025

[24] Aujla SJ, Chan YR, Zheng M, Fei M, Askew DJ, et al (2008) IL-22 mediates mucosal host defense against Gram-negative bacterial pneumonia. Nat Med 14: 275–281. 10.1038/nm1710 18264110 PMC2901867

[25] Kelly MN, Kolls JK, Happel K, Schwartzman JD, Schwarzenberger P, et al (2005) Interleukin-17/interleukin-17 receptor-mediated signaling is important for generation of an optimal polymorphonuclear response against Toxoplasma gondii infection. Infection and immunity 73: 617–621. 15618203 10.1128/IAI.73.1.617-621.2005 PMC538931

[26] Huang W, Na L, Fidel PL, Schwarzenberger P (2004) Requirement of interleukin-17A for systemic anti-Candida albicans host defense in mice. The Journal of infectious diseases 190: 624–631. 15243941 10.1086/422329

[27] Higgins SC, Jarnicki AG, Lavelle EC, Mills KH (2006) TLR4 mediates vaccine-induced protective cellular immunity to Bordetella pertussis: role of IL-17-producing T cells. Journal of immunology 177: 7980–7989. 10.4049/jimmunol.177.11.7980 17114471

[28] Rudner XL, Happel KI, Young EA, Shellito JE (2007) Interleukin-23 (IL-23)-IL-17 cytokine axis in murine Pneumocystis carinii infection. Infection and immunity 75: 3055–3061. 17403873 10.1128/IAI.01329-06 PMC1932856

[29] Brenchley JM, Paiardini M, Knox KS, Asher AI, Cervasi B, et al (2008) Differential Th17 CD4 T-cell depletion in pathogenic and nonpathogenic lentiviral infections. Blood 112: 2826–2835. 10.1182/blood-2008-05-159301 18664624 PMC2556618

[30] Micci L, Cervasi B, Ende ZS, Iriele RI, Reyes-Aviles E, et al (2012) Paucity of IL-21-producing CD4(+) T cells is associated with Th17 cell depletion in SIV infection of rhesus macaques. Blood 120: 3925–3935. 10.1182/blood-2012-04-420240 22990011 PMC3496953

[31] Pallikkuth S, Micci L, Ende ZS, Iriele RI, Cervasi B, et al (2013) Maintenance of intestinal Th17 cells and reduced microbial translocation in SIV-infected rhesus macaques treated with interleukin (IL)-21. PLoS pathogens 9: e1003471 10.1371/journal.ppat.1003471 23853592 PMC3701718

[32] Cecchinato V, Trindade CJ, Laurence A, Heraud JM, Brenchley JM, et al (2008) Altered balance between Th17 and Th1 cells at mucosal sites predicts AIDS progression in simian immunodeficiency virus-infected macaques. Mucosal immunology 1: 279–288. 10.1038/mi.2008.14 19079189 PMC2997489

[33] Raffatellu M, Santos RL, Verhoeven DE, George MD, Wilson RP, et al (2008) Simian immunodeficiency virus-induced mucosal interleukin-17 deficiency promotes Salmonella dissemination from the gut. Nat Med 14: 421–428. 10.1038/nm1743 18376406 PMC2901863

[34] Klatt NR, Estes JD, Sun X, Ortiz AM, Barber JS, et al (2012) Loss of mucosal CD103+ DCs and IL-17+ and IL-22+ lymphocytes is associated with mucosal damage in SIV infection. Mucosal immunology 5: 646–657. 10.1038/mi.2012.38 22643849 PMC3443541

[35] L. Micci, E. Ryan, C. McGary, S. Paganini, G. Silvestri, et al. (2015) IL-21 Reduces Inflammation and Virus Persistence in ART-Treated SIV-Infected Macaques. Conference on Retroviruses and Opportunistic Infections (CROI). Seattle, Washington.

[36] Klatt NR, Chomont N, Douek DC, Deeks SG (2013) Immune activation and HIV persistence: implications for curative approaches to HIV infection. Immunological reviews 254: 326–342. 10.1111/imr.12065 23772629 PMC3694608

[37] Macal M, Sankaran S, Chun TW, Reay E, Flamm J, et al (2008) Effective CD4+ T-cell restoration in gut-associated lymphoid tissue of HIV-infected patients is associated with enhanced Th17 cells and polyfunctional HIV-specific T-cell responses. Mucosal immunology 1: 475–488. 10.1038/mi.2008.35 19079215

[38] Brenchley JM, Paiardini M (2011) Immunodeficiency lentiviral infections in natural and non-natural hosts. Blood 118: 847–854. 10.1182/blood-2010-12-325936 21505193 PMC3148166

[39] Favre D, Lederer S, Kanwar B, Ma ZM, Proll S, et al (2009) Critical loss of the balance between Th17 and T regulatory cell populations in pathogenic SIV infection. PLoS pathogens 5: e1000295 10.1371/journal.ppat.1000295 19214220 PMC2635016

[40] Salgado M, Rallon NI, Rodes B, Lopez M, Soriano V, et al (2011) Long-term non-progressors display a greater number of Th17 cells than HIV-infected typical progressors. Clinical immunology 139: 110–114. 10.1016/j.clim.2011.02.008 21367666

[41] Brandt L, Benfield T, Mens H, Clausen LN, Katzenstein TL, et al (2011) Low level of regulatory T cells and maintenance of balance between regulatory T cells and TH17 cells in HIV-1-infected elite controllers. Journal of acquired immune deficiency syndromes 57: 101–108. 10.1097/QAI.0b013e318215a991 21407087

[42] Ciccone EJ, Greenwald JH, Lee PI, Biancotto A, Read SW, et al (2011) CD4+ T cells, including Th17 and cycling subsets, are intact in the gut mucosa of HIV-1-infected long-term nonprogressors. Journal of virology 85: 5880–5888. 10.1128/JVI.02643-10 21471231 PMC3126290

[43] Hartigan-O'Connor DJ, Abel K, Van Rompay KK, Kanwar B, McCune JM (2012) SIV replication in the infected rhesus macaque is limited by the size of the preexisting TH17 cell compartment. Science translational medicine 4: 136ra169. 10.1126/scitranslmed.3003941 PMC3520607 22649090

[44] Kim CJ, McKinnon LR, Kovacs C, Kandel G, Huibner S, et al (2013) Mucosal Th17 cell function is altered during HIV infection and is an independent predictor of systemic immune activation. Journal of immunology 191: 2164–2173. 10.4049/jimmunol.1300829 23894197

[45] Schuetz A, Deleage C, Sereti I, Rerknimitr R, Phanuphak N, et al (2014) Initiation of ART during early acute HIV infection preserves mucosal Th17 function and reverses HIV-related immune activation. PLoS pathogens 10: e1004543 10.1371/journal.ppat.1004543 25503054 PMC4263756

[46] Kok A, Hocqueloux L, Hocini H, Carriere M, Lefrou L, et al (2015) Early initiation of combined antiretroviral therapy preserves immune function in the gut of HIV-infected patients. Mucosal immunology 8: 127–140. 10.1038/mi.2014.50 24985081

[47] Boniface K, Blumenschein WM, Brovont-Porth K, McGeachy MJ, Basham B, et al (2010) Human Th17 cells comprise heterogeneous subsets including IFN-gamma-producing cells with distinct properties from the Th1 lineage. Journal of immunology 185: 679–687. 10.4049/jimmunol.1000366 20511558

[48] Shi G, Cox CA, Vistica BP, Tan C, Wawrousek EF, et al (2008) Phenotype switching by inflammation-inducing polarized Th17 cells, but not by Th1 cells. Journal of immunology 181: 7205–7213. 10.4049/jimmunol.181.10.7205 PMC2665021 18981142

[49] Nistala K, Adams S, Cambrook H, Ursu S, Olivito B, et al (2010) Th17 plasticity in human autoimmune arthritis is driven by the inflammatory environment. Proceedings of the National Academy of Sciences of the United States of America 107: 14751–14756. 10.1073/pnas.1003852107 20679229 PMC2930428

[50] Sandler NG, Wand H, Roque A, Law M, Nason MC, et al (2011) Plasma levels of soluble CD14 independently predict mortality in HIV infection. The Journal of infectious diseases 203: 780–790. 10.1093/infdis/jiq118 21252259 PMC3071127

[51] Burdo TH, Lentz MR, Autissier P, Krishnan A, Halpern E, et al (2011) Soluble CD163 made by monocyte/macrophages is a novel marker of HIV activity in early and chronic infection prior to and after anti-retroviral therapy. The Journal of infectious diseases 204: 154–163. 10.1093/infdis/jir214 21628670 PMC3105035

[52] Leeansyah E, Malone DF, Anthony DD, Sandberg JK (2013) Soluble biomarkers of HIV transmission, disease progression and comorbidities. Current opinion in HIV and AIDS 8: 117–124. 10.1097/COH.0b013e32835c7134 23274365

[53] Paiardini M (2010) Th17 cells in natural SIV hosts. Current opinion in HIV and AIDS 5: 166–172. 10.1097/COH.0b013e328335c161 20543595

[54] Deeks SG, Tracy R, Douek DC (2013) Systemic effects of inflammation on health during chronic HIV infection. Immunity 39: 633–645. 10.1016/j.immuni.2013.10.001 24138880 PMC4012895

[55] Paiardini M, Cervasi B, Reyes-Aviles E, Micci L, Ortiz AM, et al (2011) Low levels of SIV infection in sooty mangabey central memory CD4(+) T cells are associated with limited CCR5 expression. Nat Med 17: 830–U197. 10.1038/nm.2395 21706028 PMC3253129

[56] Micci L, Alvarez X, Iriele RI, Ortiz AM, Ryan ES, et al (2014) CD4 Depletion in SIV-Infected Macaques Results in Macrophage and Microglia Infection with Rapid Turnover of Infected Cells. PLoS pathogens 10: e1004467 10.1371/journal.ppat.1004467 25356757 PMC4214815

[57] Amara RR, Villinger F, Altman JD, Lydy SL, O'Neil SP, et al (2001) Control of a mucosal challenge and prevention of AIDS by a multiprotein DNA/MVA vaccine. Science 292: 69–74. 11393868 10.1126/science.1058915

[58] Vandergeeten C, Fromentin R, Merlini E, Lawani MB, DaFonseca S, et al (2014) Cross-clade ultrasensitive PCR-based assays to measure HIV persistence in large-cohort studies. Journal of virology 88: 12385–12396. 10.1128/JVI.00609-14 25122785 PMC4248919

[59] Nishimura Y, Sadjadpour R, Mattapallil JJ, Igarashi T, Lee W, et al (2009) High frequencies of resting CD4+ T cells containing integrated viral DNA are found in rhesus macaques during acute lentivirus infections. Proceedings of the National Academy of Sciences of the United States of America 106: 8015–8020. 10.1073/pnas.0903022106 19416840 PMC2683103

[60] Hansen SG, Piatak M Jr., Ventura AB, Hughes CM, Gilbride RM, et al (2013) Immune clearance of highly pathogenic SIV infection. Nature 502: 100–104. 10.1038/nature12519 24025770 PMC3849456

