# Supplementary figures and images for: Loss of Function of Intestinal IL-17 and IL-22 Producing Cells Contributes to Inflammation and Viral Persistence in SIV-Infected Rhesus Macaques
Source: PLoS Pathog. 2016 Feb 1;12(2):e1005412. doi: 10.1371/journal.ppat.1005412 (PMC4735119; doi:10.1371/journal.ppat.1005412)

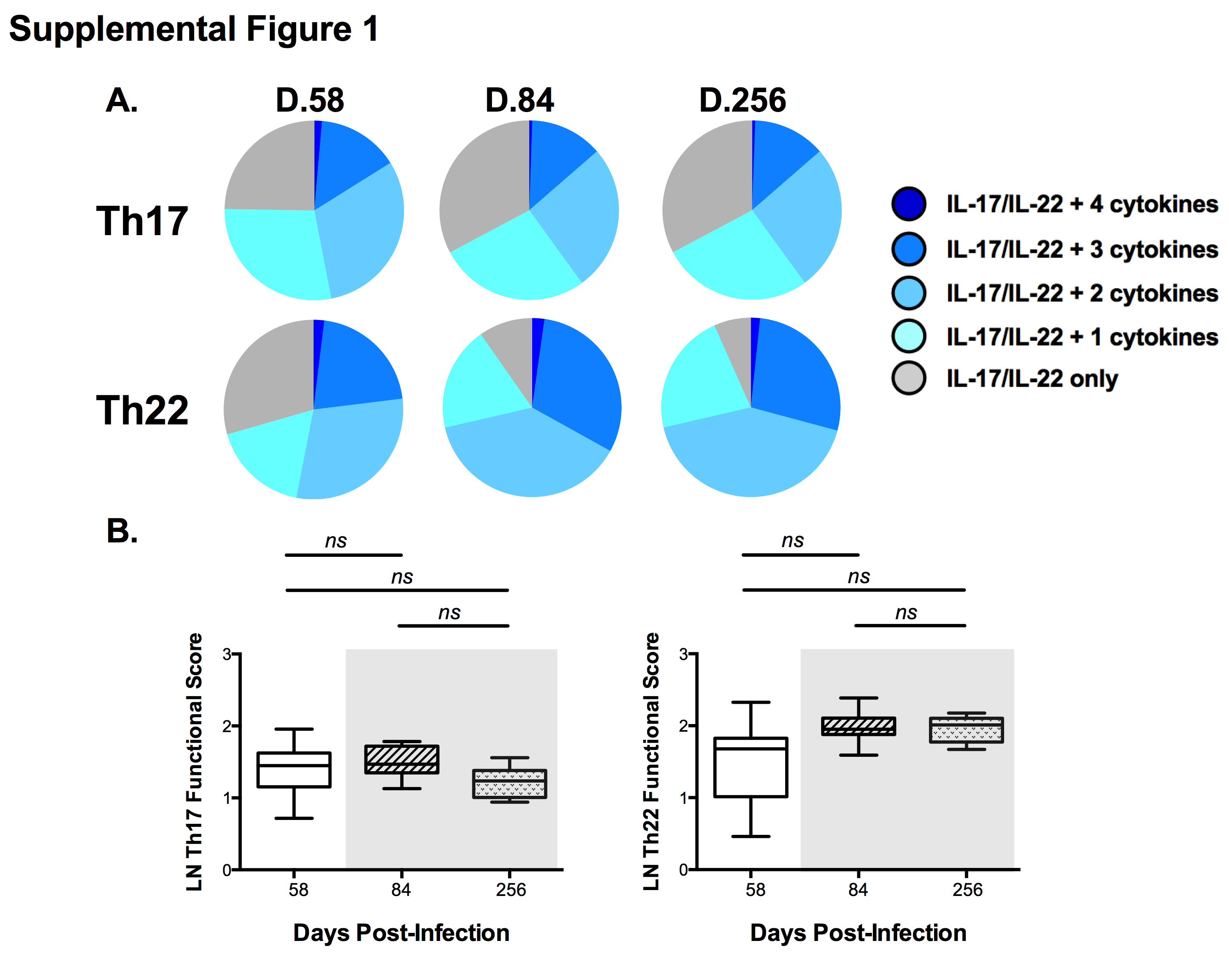

Supplement: S1 Fig — Longitudinal assessment of LN Th17 and Th22 cytokine profiles (A) and functional score (B) at SIV infection (d. 58 p.i.), early ART treatment (d. 84 p.i.), and late ART treatment (d. 256 p.i.). Both cytokine profiles and functional score remained statistically unchanged during ART. Dotted line marks time of SIV infection and shaded gray box represents time of ART treatment. Averaged data are presented as box and whisker plots, with the median functional score in between the 25% and 75% quartiles. (TIFF) [file ppat.1005412.s001.tiff]

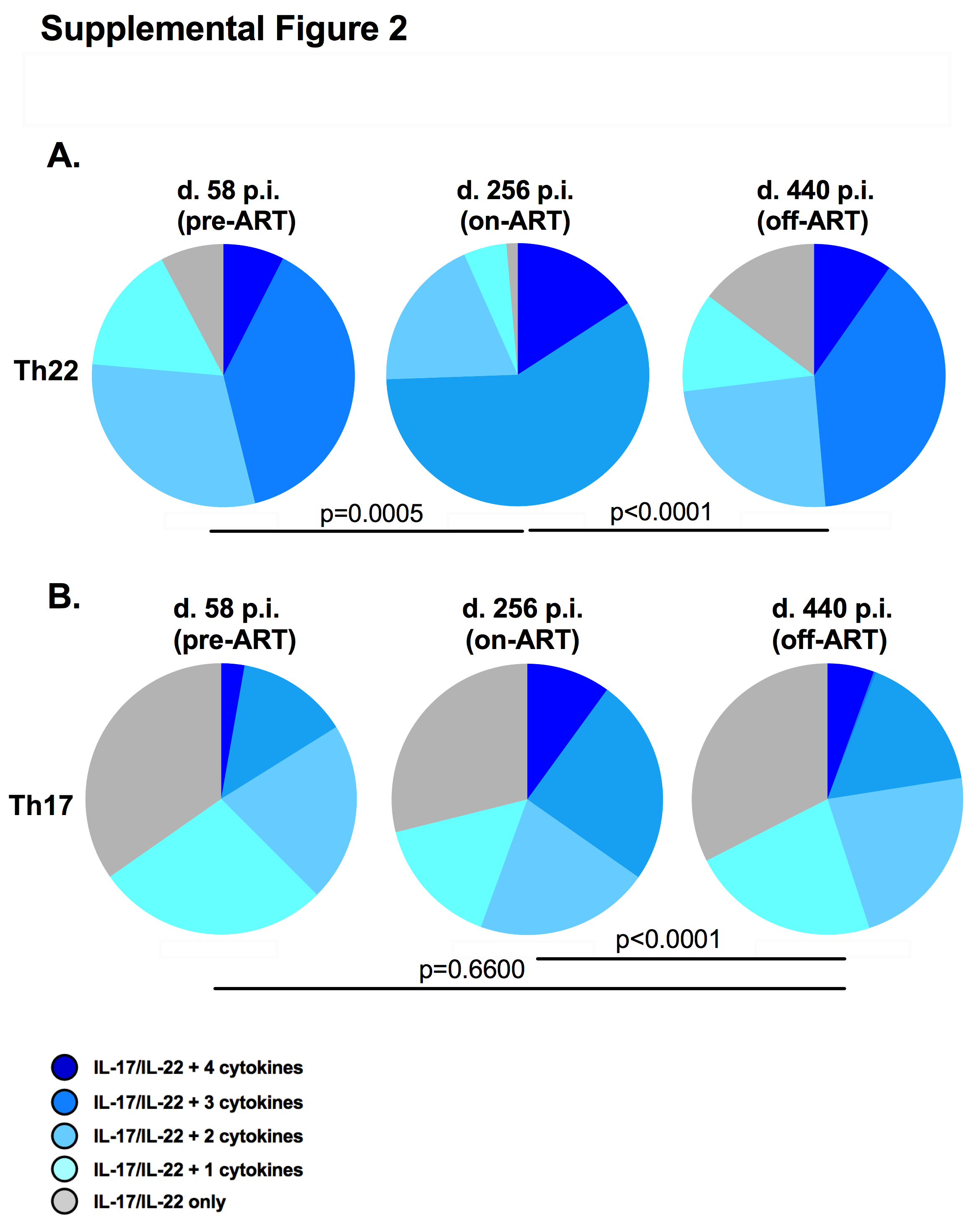

Supplement: S2 Fig — Longitudinal assessment of intestinal Th22 (A) and Th17 (B) cytokine profiles during chronic SIV infection (d. 58 p.i.), late ART treatment (d. 256 p.i.) and at six months after ART interruption (d. 440 p.i.). Cytokine profiles were generated for each cell population by SPICE program v. 5.33, and were calculated by Flowjo Boolean gating. (TIFF) [file ppat.1005412.s002.tiff]

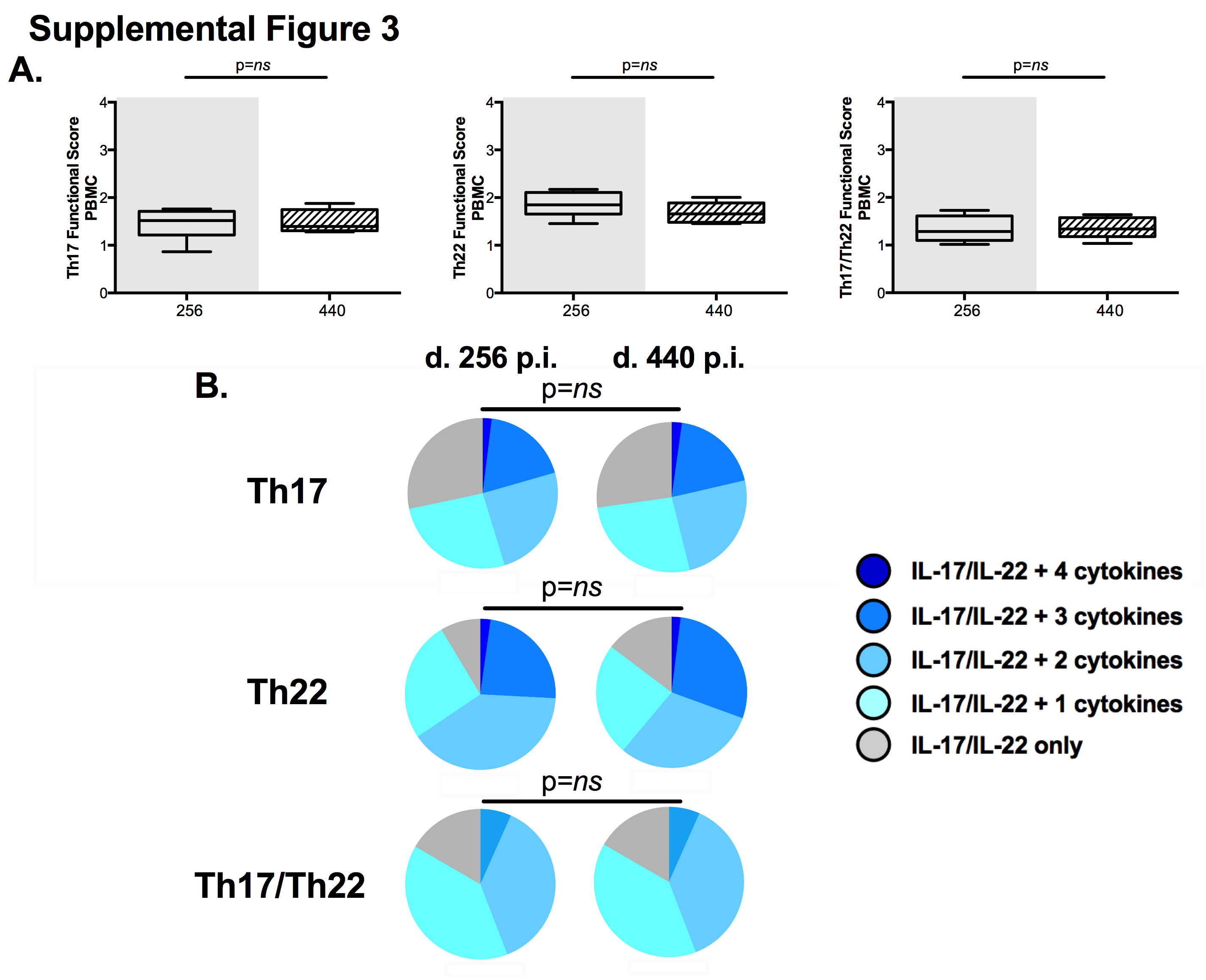

Supplement: S3 Fig — Comparison of blood Th17, Th22 and Th17/Th22 functional score (A) and cytokine profiles (B) between pre (d. 256 p.i.) and post-ART interruption (d. 440 p.i.). Both cytokine profiles and functional score remained statistically unchanged before and after ART discontinuation. Shaded gray box represents time of ART treatment. Averaged data are presented as box and whisker plots, with the median functional score in between the 25% and 75% quartiles. Cytokine profiles were generated for each cell population by SPICE program v. 5.33, and were calculated by Flowjo Boolean gating. (TIFF) [file ppat.1005412.s003.tiff]

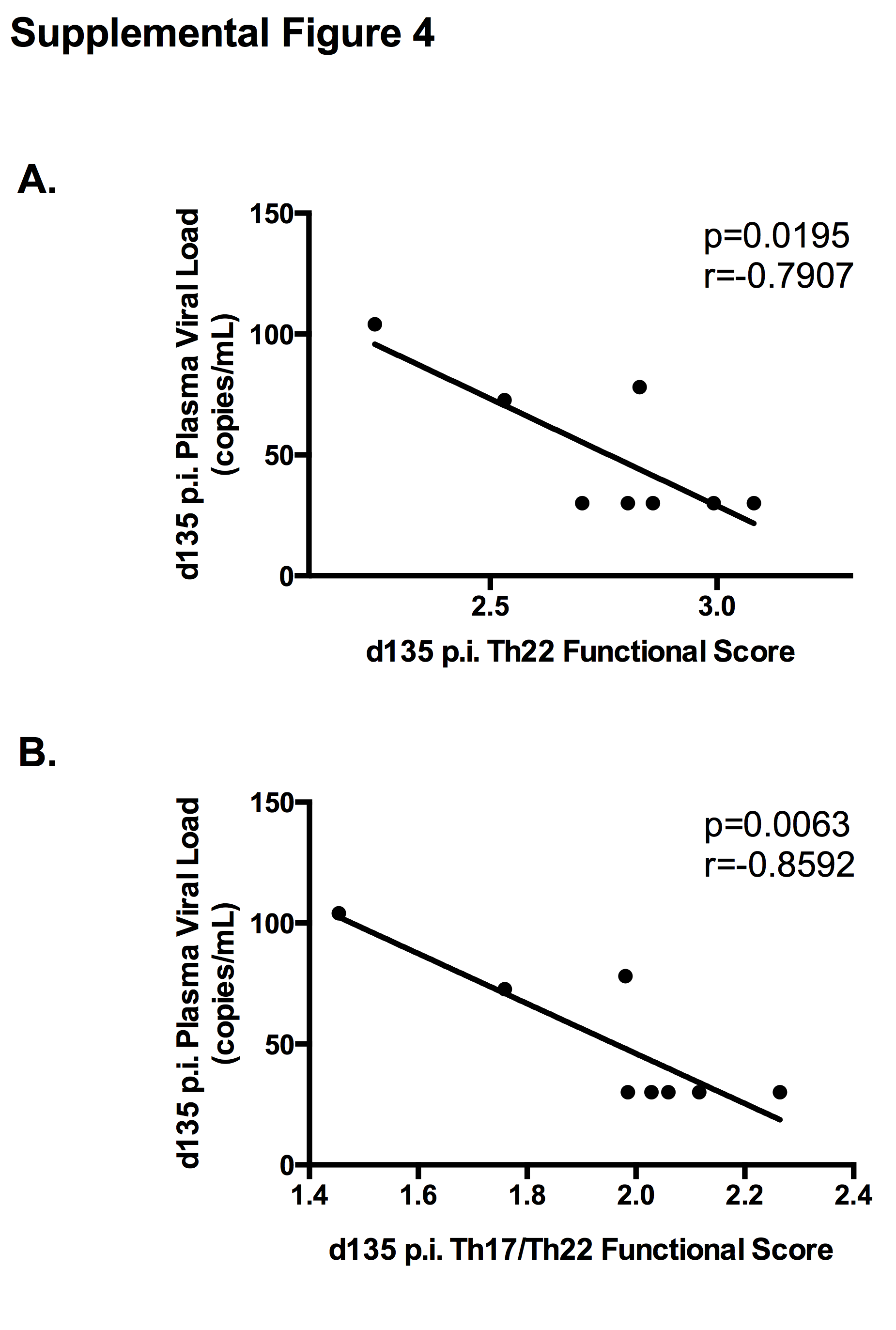

Supplement: S4 Fig — Intestinal Th22 (A) and Th17/Th22 (B) functional scores at d135 p.i. inversely correlate with plasma viral load levels at the same experimental point (d. 135 p.i.). (TIFF) [file ppat.1005412.s004.tiff]

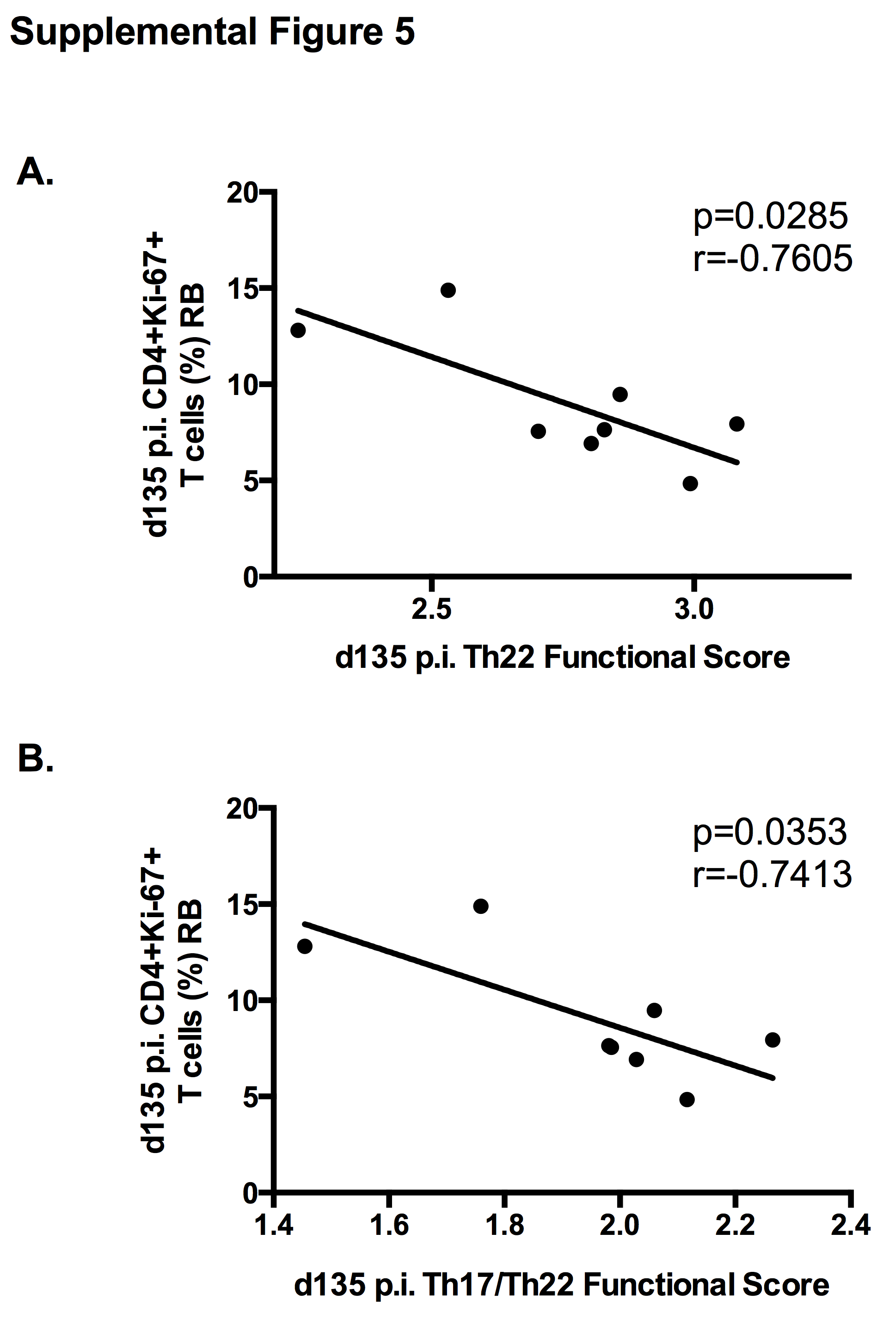

Supplement: S5 Fig — Intestinal Th22 (A) and Th17/Th22 (B) functional scores at d135 p.i. negatively correlate with intestinal CD4+ T cell proliferation levels (Ki-67+). (TIFF) [file ppat.1005412.s005.tiff]

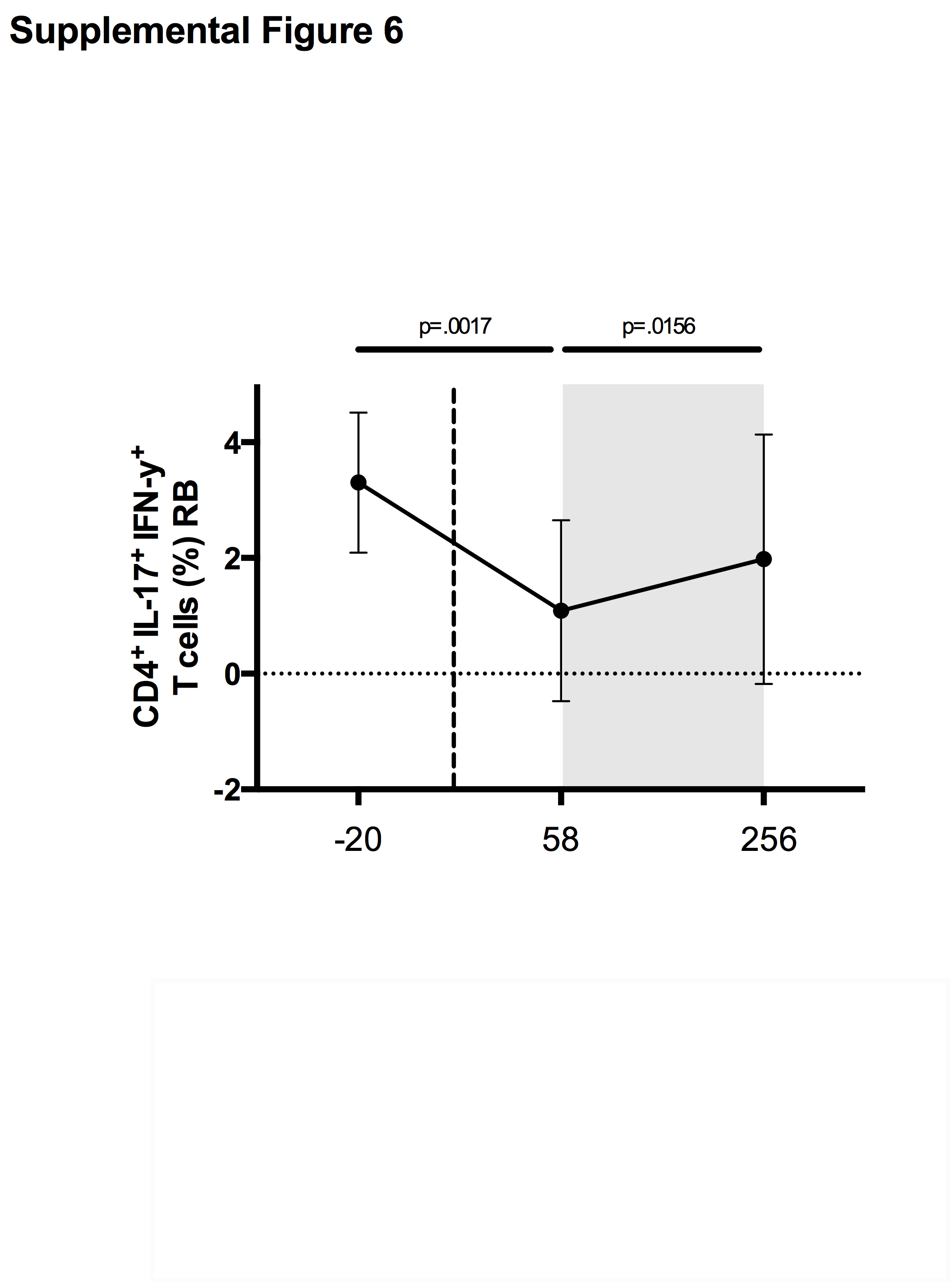

Supplement: S6 Fig — Intestinal IL-17+IFN-γ+ CD4+ T cells are significantly depleted during chronic SIV infection and not fully restored during the 7 months of ART treatment. Dotted line marks time of SIV infection and shaded gray box represents time of ART treatment. Averaged data are presented as means with SD. (TIFF) [file ppat.1005412.s006.tiff]

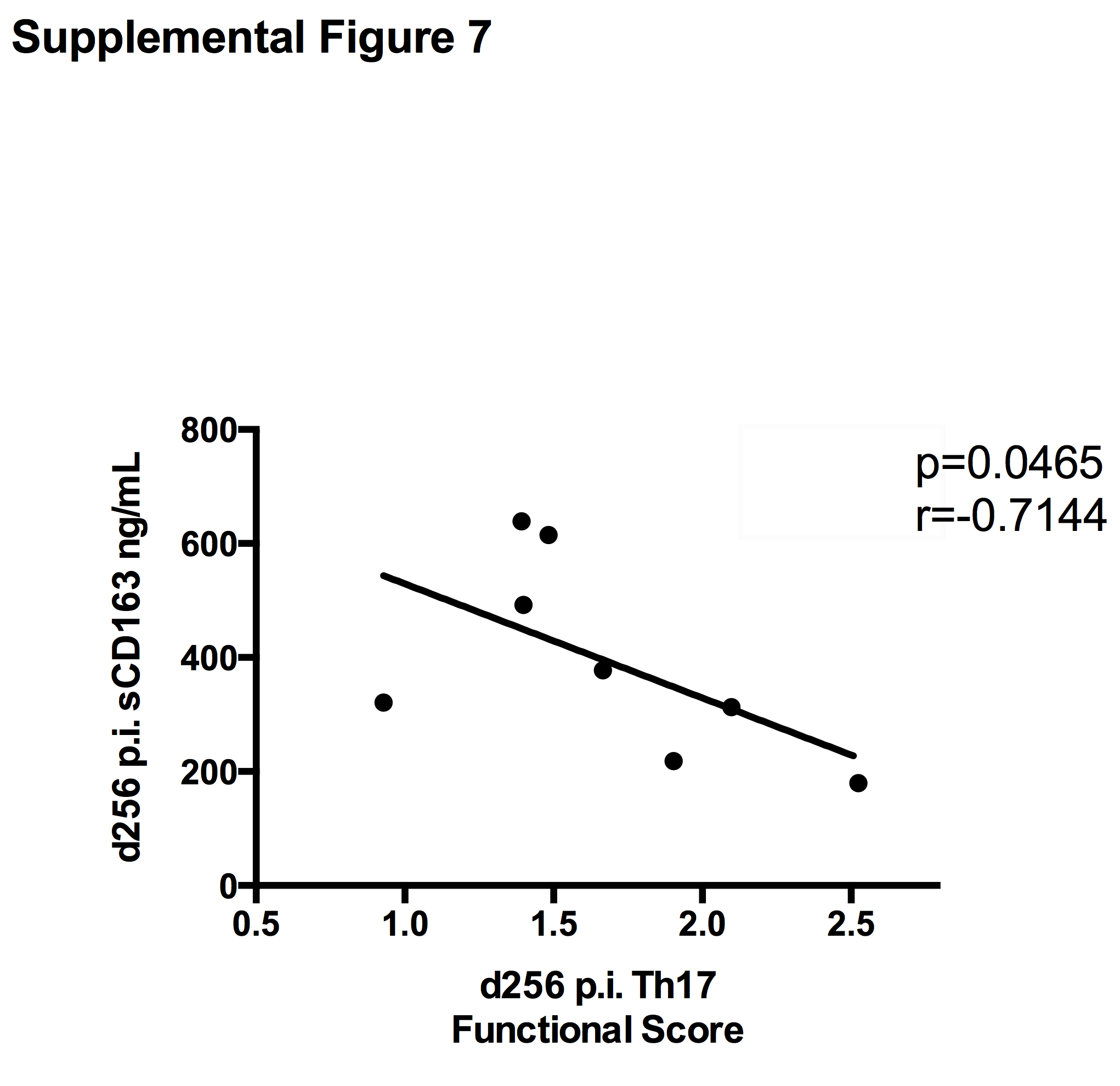

Supplement: S7 Fig — Higher functional scores of Th17 cells at d256 p.i. negatively correlated with levels of sCD163 at the same experimental point. (TIFF) [file ppat.1005412.s007.tiff]

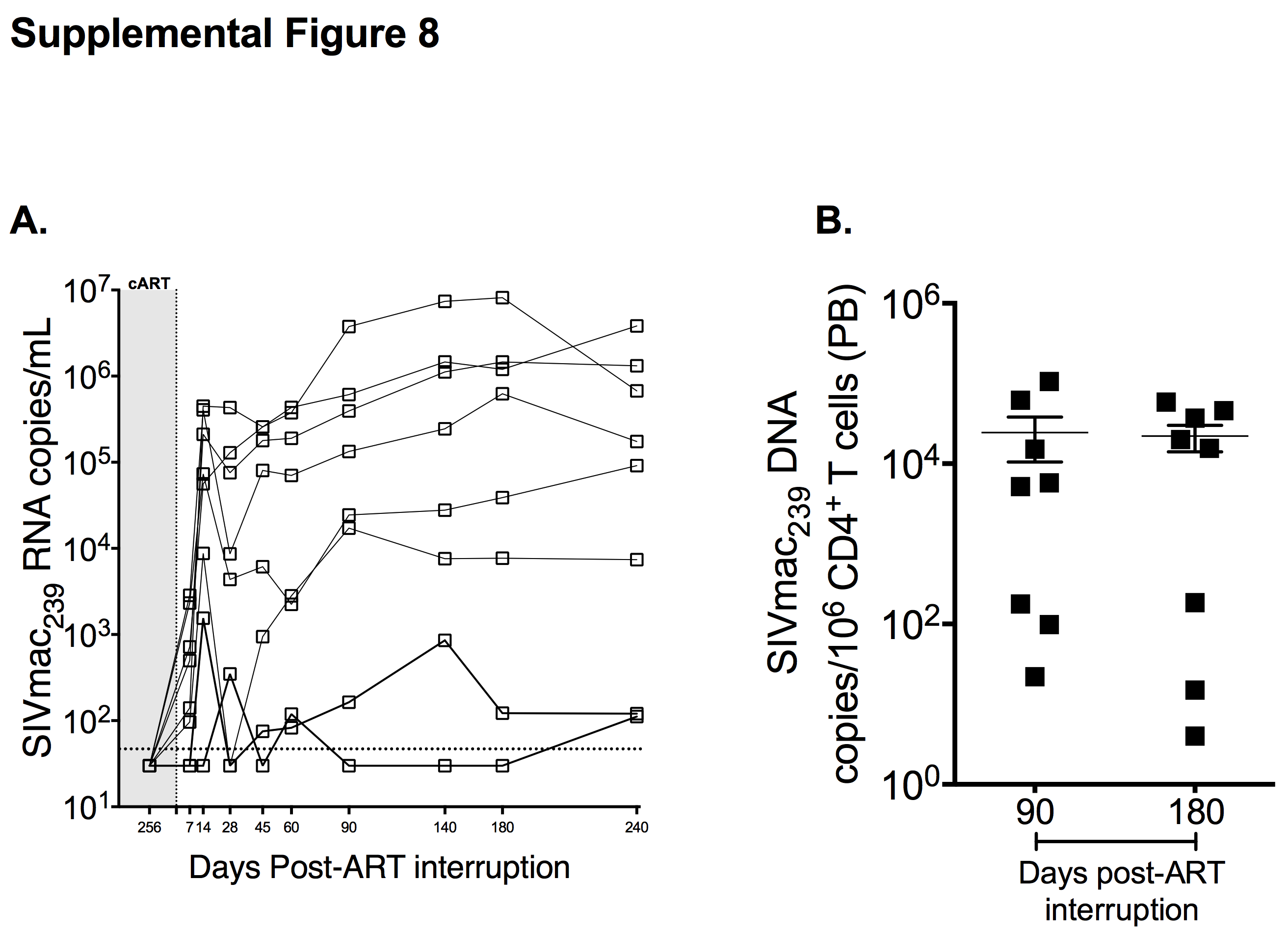

Supplement: S8 Fig — (A) Plasma levels of SIVmac239 RNA, expressed as copies/ml and (B) peripheral blood SIVmac239 DNA content, expressed as copies/1,000,000 CD4+ T-cells, are shown in 8 RMs that underwent structured ART interruption. (TIFF) [file ppat.1005412.s008.tiff]
