# Supplementary material for: Loss of Function of Intestinal IL-17 and IL-22 Producing Cells Contributes to Inflammation and Viral Persistence in SIV-Infected Rhesus Macaques
Source: PLoS Pathog. 2016 Feb 1;12(2):e1005412. doi: 10.1371/journal.ppat.1005412 (PMC4735119; doi:10.1371/journal.ppat.1005412)
Supplement: S1 Table — The relationship between intestinal SIV-DNA levels and Th17 and Th22 cell function at d. 256 p.i. was conducted with adjustment for pre-ART (d.58 p.i.) plasma SIVmac239 levels. Adjusted linear regressions models for both subsets were run with the sample size of 8 animals. (DOCX) [file ppat.1005412.s009.docx]

**Supplemental Table 1**. **Linear regression adjusted for baseline plasma viremia. Sample size = 8**

| Outcome | Predictors, X1 | Predictors, X2 | Regression Equation | | Mean Square Error | | X1 Slope ± SE | P value for slope = 0 | X2 Slope ± SE | P value for slope = 0 | r^2^,r | Adjusted r^2^ | Fig 8 |
| --- | --- | --- | --- | --- | --- | --- | --- | --- | --- | --- | --- | --- | --- |
| D. 256 p.i. SIV-DNA | D. 256 p.i. Th17 function |  | | Ŷ = 4.878 – 1.0695X1 + 0.13747X2 | | 0.0382 | -1.0695 ± 0.15005 | 0.0008 | - | - | 0.92,.96 | 0.88 | **8A** |
|  |  | D. 58 p.i. VL | | - | | - | - | - | 0.13747 ± 0.0657 | 0.09 | - | - |  |
| D. 256 p.i. SIV-DNA | D. 256 p.i. Th22 function |  | | Ŷ = 10.558 – 2.74497X1 + 0.20518X2 | | 0.07105 | -2.74497 ± 0.5489 | 0.004 | - | - | 0.84,.92 | 0.78 | **8B** |
|  |  | D. 58 p.i. VL | | - | | - | - | - | 0.20518 ± 0.0914 | 0.08 | - | - |  |
